# Histone Deacetylase Inhibitor Improves the Dysfunction of Hippocampal Gamma Oscillations and Fast Spiking Interneurons in Alzheimer’s Disease Model Mice

**DOI:** 10.3389/fnmol.2021.782206

**Published:** 2021-12-23

**Authors:** Keiko Takasu, Kazuki Niidome, Minoru Hasegawa, Koichi Ogawa

**Affiliations:** Pain and Neuroscience, Drug Discovery and Disease Research Laboratory, Shionogi & Co., Ltd., Osaka, Japan

**Keywords:** histone deacetylase (HDAC), gamma oscillation, fast spiking interneurons, hippocampus, Alzheimer’s disease

## Abstract

The hippocampal gamma oscillation is important for cognitive function, and its deficit is related to cognitive impairment in Alzheimer’s disease (AD). Recently, it has been recognized that post-translational modification via histone acetylation is a fundamental molecular mechanism for regulating synaptic plasticity and cognitive function. However, little is known regarding the regulation of hippocampal gamma oscillation by histone acetylation. We investigated whether histone acetylation regulated kainate-induced gamma oscillations and their important regulator, fast-spiking interneurons, using acute hippocampal slices of AD model mice (PSAPP transgenic mice). We found a decrease in kainate-induced gamma oscillations in slices from PSAPP mice, accompanied with the increased activity of fast spiking interneurons in basal state and the decreased activity in activated state. The histone deacetylase (HDAC) inhibitor (SAHA, named vorinostat) restored deficits of gamma oscillation in PSAPP mice, accompanied with rescue of activity of fast spiking interneurons in basal and activated state. The effect of SAHA was different from that of the clinical AD drug donepezil, which rescued only function of fast spiking interneurons in basal state. Besides, activator of nuclear receptor family 4a (NR4a) receptor (cytosporone B), as one of the epigenetic modification related to HDAC inhibition, rescued the deficits in gamma oscillations in PSAPP mice. These results suggested a novel mechanism in which HDAC inhibition improved impairment of gamma oscillations in PSAPP mice by restoring the activity of fast spiking interneurons both in basal and activated state. The reversal of gamma oscillation deficits by HDAC inhibition and/or NR4a activation appears to be a potential therapeutic target for treating cognitive impairment in AD patients.

## Introduction

Alzheimer’s disease (AD) is the most common form of dementia, and patients with the disease experience progressive cognitive dysfunction (Selkoe, 2002; Walsh and Selkoe, 2007). This dysfunction is caused by aberrant amyloid beta formation (Selkoe, 2002; Walsh and Selkoe, 2007; Palop and Mucke, 2010). Over the past several decades, most studies have focused on the effects of amyloid beta on the neuronal excitability and synaptic function of single neurons, including the ion channel properties of individual cells (Hartley et al., 1999; Kamenetz et al., 2003; Ye et al., 2003). However, it has recently become clear that understanding the influence of amyloid beta and its downstream molecular signals on the progressive alteration of synaptic function and neuronal network activity between multiple neurons is important for understanding cognitive dysfunction (Palop and Mucke, 2010). This is supported by evidence from multi-neuronal network analysis, including EEG recordings, in which both clinical patients and an animal model of AD exhibit cognitive dysfunction associated with aberrant neuronal network activity in the hippocampus and cortex (Herrmann and Demiralp, 2005; Verret et al., 2012; Hazra et al., 2013; Born et al., 2014; Igarashi et al., 2014). To develop an effective treatment for cognitive impairment, it is important to understand the pathological mechanisms of AD that are related to multi-neuronal network activity.

Oscillatory activity is a hallmark of neuronal network function in various brain regions, including the hippocampus and cortex (Bartos et al., 2007; Buzsaki and Wang, 2012; Iwasaki et al., 2021). In particular, gamma oscillations are considered to be involved in cognitive function and memory processes such as encoding and recall in both animals (Yamamoto et al., 2014) and humans (Fell et al., 2001; Sederberg et al., 2007; Herrmann et al., 2010). Gamma oscillations are thought to be controlled mainly by fast-spiking, parvalbumin (PV)-expressing GABA-containing interneurons (Bartos et al., 2007). In various psychiatric disorder animal models, such as AD or schizophrenia, disruption of gamma oscillations related to an inhibitory interneuron and/or excitatory-inhibitory imbalance might underlie cognitive dysfunction (Herrmann and Demiralp, 2005; Lewis et al., 2012; Verret et al., 2012). Understanding the mechanisms and functions underlying gamma oscillation in a pathological state is necessary to understand abnormal memory processes and can yield therapeutic opportunities for cognitive dysfunction.

Recent studies have suggested that the post-translational modification of histone proteins and the subsequent alterations in gene expression could be a fundamental molecular mechanism for the regulation of synaptic plasticity and memory formation (Gräff and Tsai, 2013; Mews et al., 2021). In particular, histone acetylation has been the subject of many studies because histone deacetylase (HDAC) inhibitors enhance both memory and synaptic function in AD model mice (Ricobaraza et al., 2009). Conversely, hippocampal overexpression of one HDAC family member, HDAC2, inhibits spine formation and exacerbates memory impairment in mice (Guan et al., 2009). Thus, histone acetylation is considered a therapeutic target for the treatment of cognitive impairment in AD patients. This is supported by evidence of elevated HDAC2 expression in AD patients (Gräff and Tsai, 2013). Although it is known that histone acetylation modifies the expression of a variety of genes and subsequently alters spine dynamics (Guan et al., 2002; Gräff and Tsai, 2013; Mews et al., 2021), its influence on neuronal network activity remains unclear. In particular, there is no evidence for the influence of HDAC inhibition on gamma oscillations, which are a hallmark of the neuronal network activity related to cognitive function. Thus, it is important to evaluate the functional influence of histone acetylation on aberrant neuronal network activity in the pathological state.

In the present study, we demonstrated that the gamma oscillation induced by the application of kainate was attenuated in hippocampal slices prepared from AD model mice (PSAPP transgenic mice), and application of donepezil, a clinically used drug for AD treatment, recovered the gamma oscillation. We demonstrated for the first time that HDAC inhibitor suberoylanilide hydroxamic acid (SAHA) elevated the gamma oscillation level, and the effect of SAHA was accompanied with the elevation of histone H3 and H4 acetylation. The effects of SAHA on the gamma oscillation and histone acetylation were abolished by co-application of the histone acetyltransferase (HAT) inhibitor C646, showing that histone acetylation is a key mechanism for the regulation of gamma oscillations by SAHA. Interestingly, SAHA and donepezil, differently rescued activity of fast spiking interneurons in basal and activated state. These results indicated a novel mechanism in which HDAC inhibition improved impairment of gamma oscillations in PSAPP mice by restoring activity of fast spiking interneurons both in basal and activated state.

## Materials and Methods

### Animals

Experiments were performed using double-transgenic mice (Tg-APP/PS1, referred to as PSAPP mice, male). PSAPP mice were generated by crossing presenilin1 “knock-in” mice (named PS mice; Nakano et al., 1999) with transgenic mice (Tg2576, Taconic Biosciences, Inc., United States) that overexpress the Swedish mutation of the human amyloid precursor protein (APP; Hsiao et al., 1996). The breeding of PSAPP and PS mice was approved by the Animal Care and Use Committee of Shionogi Research Laboratories and was in accordance with the Association for Assessment and Accreditation of Laboratory Animal Care (AAALAC) International guidelines. C57BL/6J Jcl mice (male) were purchased from CLEA Japan, Inc. and were used as wild-type controls. Body weights of mice were 25–50 g. Mice aged at 3 or 14 months were used. The mice were housed under controlled temperature and humidity with a 12/12-h light/dark cycle. Less than three mice were housed in a cage (W 235 mm, D 353 mm, H 160 mm) with bedding paper chips (SLC Japan, Inc.) and Nesting Sheets™ (Bio-Serv, United States) under environmental enrichment. Mice were allowed *ad libitum* access to food (CE-2, CLEA Japan, Inc.) and clean water (filtered at 5 μm from Toyonaka city, Japan) under SPF conditions. All procedures were approved by the Animal Care and Use Committee of Shionogi Research Laboratories, Osaka, Japan. Electrophysiological assessments were performed according to the AAALAC International guidelines.

### Experimental Procedures

For preparation of slices, mice were euthanized by cervical dislocation not under anesthesia. Well trained experimenters with advanced animal handling skills performed this procedure, trying to minimize stress to the mice and carefully observing the condition of the mice during the restraint.

#### Interpretation

All studies involving animals were performed in accordance with the ARRIVE guidelines for reporting experiments involving animals (Kilkenny et al., 2010; McGrath et al., 2010). The present study was performed according to the 3Rs.

#### Group Sizes

We described the *n* value of each group in the figure legends. For data subjected to statistical analysis, the sample size was more than 5. Group size was not extremely varied. We did not exclude data except for the predetermined exclusion criteria: stable recordings were not maintained due to the slice condition and/or changes in the recording conditions.

#### Randomization

A valid scientific justification for correcting randomized data was determined by the following method: some pieces of horizontal slices prepared from the dorsal to ventral hippocampus of a mouse were utilized following randomization. Then, we treated the slices with drug according to a predetermined drug application protocol before beginning of the recording. We did not change the drug application protocol during experiments.

#### Blinding

The operator and data analyst was the same person and so blinding was not undertaken. However, a valid scientific justification was determined by the following method: (1) Transformation of raw data into values was performed automatically by a programmed algorithm in NeuroExplorer software (Nex Technologies, United States) or the Mini Analysis Program (Synaptosoft Inc., Decatur, GA, United States). Thus, the value was calculated according to predetermined criteria in the software program, and biased interpretations could not appear. (2) The value was calculated in a state in which the drug condition of the data (vehicle or drug-treated) was not known until finally summarizing the data. The details of the data (vehicle or drug-treated) were disclosed when the final data were averaged. This procedure was also performed automatically according to the program, therefore, there was no bias in averaging the data. (3) Reliable statistical analyses were undertaken automatically by a programmed algorithm using analysis software with GraphPad Prism (GraphPad Software, Inc. United States).

#### Normalization

We did not perform normalization of the data directly used for the parametric statistical analysis. For the calculation of power spectral densities (PSD), values were normalized to the total PSD between 0 and 50 Hz using refined analysis software with NeuroExplorer (Nex Technologies). This normalization was performed uniformly under predetermined criteria by an automated refined program and did not directly influence statistical significance.

### Data and Statistical Analysis

Differences between groups were analyzed for statistical significance with Dunnett’s test for multiple groups and with Student’s *t*-test for two groups. Statistical analysis used independent values. *P* < 0.05 was taken to indicate statistical significance.

### Preparation of Hippocampal Slices

The mice were euthanized by cervical dislocation. The brains were then quickly removed and placed in ice-cold, low-sodium artificial CSF containing the following: 215.5 mM sucrose, 3 mM KCl, 1 mM NaH_2_PO_4_, 25 mM NaHCO_3_, 11 mM D-glucose, 1 mM CaCl_2_, and 5 mM MgCl_2_ (pH 7.4 after bubbling with 95% O_2_ and 5% CO_2_). Horizontal slices (350 μm thick) were prepared using a vibratome (VT1200S; Leica, Germany) and then maintained for at least 60 min in standard artificial CSF containing the following: 113 mM NaCl, 3 mM KCl, 1 mM NaH_2_PO_4_, 25 mM NaHCO_3_, 11 mM D-glucose, 2 mM CaCl_2_, and 1 mM MgCl_2_ (pH 7.4, after bubbling with 95% O_2_ and 5% CO_2_ at 30–32^°^C). Slices were transferred to a recording chamber mounted on the stage of a microscope (IX70; Olympus, Japan) and were superfused with standard artificial CSF (flow rate of 2.5 mL min^–1^ at 30–32^°^C).

### Electrophysiological Recording and Data Analysis for Assessment of Gamma Oscillation

#### Electrophysiological Recording

Hippocampal slices were placed in the recording chamber with a 64 planar multi-electrode array, each electrode with a diameter of 50 μm (MED-P50025; Alpha MED Scientific, Inc, Japan). Local field potentials in CA1, CA3, and DG were recorded with a multichannel recording system (Cerebus Data Acquisition System; Blackrock Microsystems, United States) equipped with a high-cut filter at 125 Hz. The signal was digitized at 10 kHz with Central software (Blackrock Microsystems, United States). To assess the effects of drugs on local field potentials, drugs were delivered for 40 min, and kainate (with drugs) was then applied for 10 min. Gamma oscillatory activity, which was detected as neuronal oscillatory activity in local field potentials at a gamma frequency of 20–40 Hz (Fisahn et al., 2004; Bartos et al., 2007; Spencer et al., 2010), was assessed for the last 3 min of the kainate treatment (10 min).

#### Data Analysis

**FIGURE 1:**
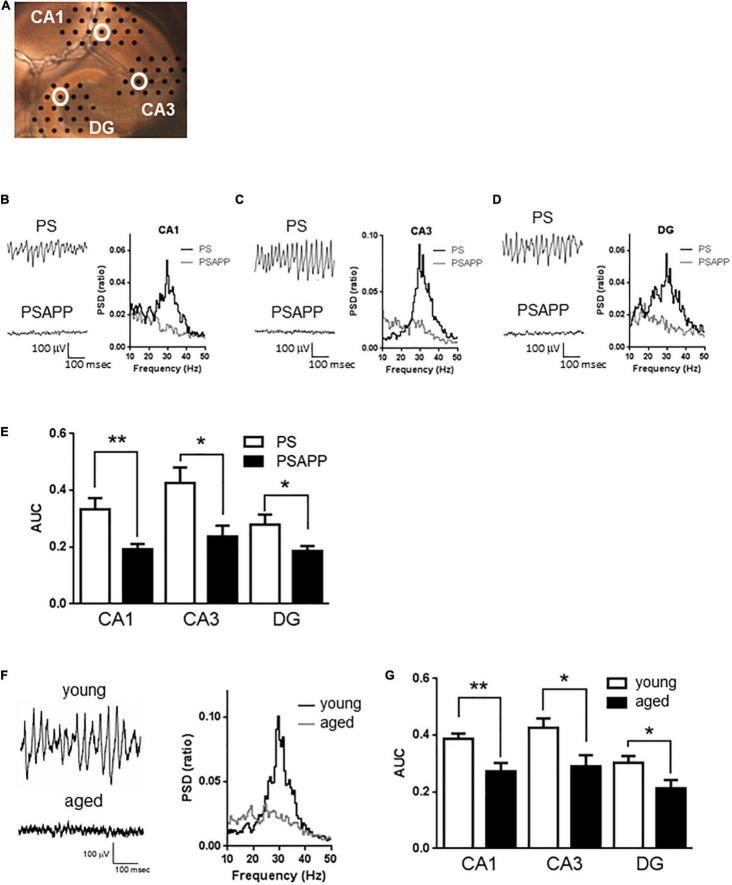
A deficit in kainate-induced gamma oscillations in the CA1, CA3, and DG of hippocampal slices prepared from PSAPP mice. **(A)** A representative image illustrating the mouse hippocampal slice and the recording electrodes (black dots). **(B–D)** Representative traces (left panel) and PSD (right panel) indicating that kainate (200 nM) strongly elicited the oscillatory activity of local field potentials at a frequency of 20–40 Hz (gamma oscillation) in PS mice but not in PSAPP mice (the recording electrodes are indicated by open white circles in **(A)**). **(E)** The average power of the kainate-induced gamma oscillations were quantified as AUC in the PSD between 20 and 40 Hz. Kainate-induced gamma oscillations were greatly attenuated in the CA1, CA3, and DG of hippocampal slices from PSAPP mice compared to those from PS mice (*p* < 0.01 at CA1, *p* < 0.05 at CA3 and DG, PS mice; *n* = 5, PSAPP mice; *n* = 7). **(F,G)** Deficits in kainate-induced gamma oscillations in the CA1, CA3, and DG in hippocampal slices prepared from aged mice. **(F)** Representative traces (left panel) and PSD (middle panel) at CA3 indicating that kainate (200 nM) strongly elicited gamma oscillation in young PS mice (3 months) while it did not occur in the aged mice (14 months). **(G)** The average power of the kainate-induced gamma oscillations was greatly attenuated in the CA1, CA3, and DG of hippocampal slices from aged PS mice compared to those of young PS mice (*p* < 0.01 at CA1, *p* < 0.05 at CA3 and DG, young mice; *n* = 8, aged mice; *n* = 9). *p < 0.05 and **p < 0.01.

The data were analyzed using NeuroExplorer software (Nex Technologies). PSD was obtained over 60-s-long recording periods using a fast Fourier Transform algorithm and were indicated as a value normalized to the total PSD between 0 and 50 Hz. The gamma power was quantified as AUC in the PSD between 20 and 40 Hz. The averaged gamma power over 3 min from each electrode in CA1, CA3, and DG (CA1: value from 23 electrodes; CA3: 19 electrodes; DG: 21 electrodes, electrode configuration is shown in Figure 1A) was calculated to assess the effects of drugs on kainate-induced gamma oscillation. Differences between groups were analyzed for statistical significance with Dunnett’s test for multiple groups and with Student’s *t*-test for two groups. *P* < 0.05 was taken to indicate statistical significance. All data are expressed as means ± SEM.

### Electrophysiological Recording and Data Analysis for Assessment of the Activity of Fast Spiking Interneurons

#### Electrophysiological Recording

Whole-cell voltage-clamp recordings were made from visually and electrophysiologically identified fast spiking interneurons in the CA1 area using an upright microscope (BX51WI, Olympus, Japan) with infrared differential interference contrast optics. The recorded neurons were located at the CA1 stratum pyramidal-oriens border and exhibited sustained fast spiking activity following current injection. Patch electrodes (2.5–3.0 μm tip diameter) were pulled from borosilicate glass capillaries and had a resistance of 3–5 MΩ when filled with an internal solution consisting of the following: 130 mM K-gluconate, 5 mM NaCl, 20 mM HEPES, 0.25 mM EGTA, 4 mM MgCl_2_, 4 mM Mg-ATP and 0.4 mM Tris–GTP, pH 7.3, adjusted with KOH. Membrane voltage was recorded with a patch clamp system (EPC-10; HEKA, Darmstadt, Germany) and PowerLab (ADInstruments, Dunedin, New Zealand), low-pass filtered at 4 kHz, and digitized at 25 kHz for computer analysis with the Pulse software (HEKA) and LabChart software (ADInstruments). All experiments were performed at 30–32^°^C. In the experiments of action potential following current injection and kainate treatment, membrane potentials were not adjusted by injecting currents and the frequency and amplitude of action potentials were assessed before and after current injection or for the last 3 min of the kainate treatment (10 min).

#### Data Analysis

The frequency and amplitude of action potentials were analyzed with the Mini Analysis Program (Synaptosoft Inc., Decatur, GA, United States). Differences between groups were analyzed for statistical significance with Dunnett’s test for multiple groups. *P* < 0.05 was taken to indicate statistical significance. All data are expressed as means ± SEM.

### Immunoblotting

Hippocampal slices were homogenized in buffer containing 50 mM Tris–HCl (pH 6.8), 150 mM NaCl, 2 mM EDTA, and 1% Triton X-100 and incubated on ice for 15 min. To isolate histones, 2 M HCl was added (final 0.2 M) and incubated on ice for 30 min. After neutralization using 1 M NaOH, the homogenate was sonicated and centrifuged at 9,300 *g* for 10 min. The supernatant was used for immunoblotting. The samples were mixed with 4 x sample buffer (Invitrogen, United States), resolved into NuPAGE Bis-Tris gels (Invitrogen, Japan), and transferred to polyvinylidene difluoride membranes (Invitrogen, United States). The membranes were blocked with blocking buffer (5% milk, 0.05% Tween in PBS) and incubated with primary antibodies (acetylated-histone H3: x1000, acetylated-histone H4: x2000; GAPDH: x500; purchased from Millipore, United States) at 4^°^C for 16 h. The membranes were washed twice in PBS containing 0.05% Tween 20, and then immunoblotted bands were detected by HRP-conjugated anti-rabbit IgG (x5000, for acetylated-histone; purchased from Jackson Immuno Research, United States) or anti-mouse IgG (x5000, for GAPDH; purchased from Jackson Immuno Research, United States) using LAS-3000 (Fujifilm, Japan). Immunoblotted bands were quantified using Multi Gauge software (Fujifilm, Japan).

### Drugs

Kainate was purchased from Tocris Bioscience (United Kingdom). SAHA (vorinostat) was purchased from Cayman Chemical Company (United States). MS-275 (entinostat) was purchased from Selleckchem (United States). Donepezil, C646 and cytosporone B were obtained from Sigma-Aldrich (St. Louis, MO, United States). Kainate and donepezil were dissolved in distilled water. SAHA, MS-275, C646 and cytosporone B were dissolved in DMSO. The drugs were administered by bath application.

## Results

### Attenuation of Gamma Oscillations in the CA1, CA3, and DG of Hippocampal Slices From PSAPP Mice

To examine whether the overexpression of APP affected hippocampal gamma oscillations, we evaluated kainate-induced gamma oscillations in slices prepared from PSAPP mice at 3 months old and compared it with those from age-matched PS and wild-type mice (C57BL/6J Jcl). In hippocampal slices of PS mice, kainate (200 nM) strongly elicited gamma oscillatory activity of local field potentials at a peak frequency of 20–40 Hz around the stratum radiatum of the CA1, CA3, and DG (Figures 1A–D). In contrast, in slices of PSAPP mice, kainate-induced gamma oscillations were not induced by kainate treatment (Figures 1B–D right panel). The PSD indicated that the peak power of approximately 20–40 Hz was strongly decreased in PSAPP mice compared with PS mice (Figures 1B–D). Figure 1E shows that PSAPP mice exhibited significant deficits in the average gamma power in the CA1, CA3, and DG compared with PS mice (Figure 1E, *p* < 0.01 at CA1, *p* < 0.05 at CA3 and DG, *n* = 5–7). Similar deficits in gamma oscillations were observed in hippocampal slices of aged PS mice at 14 months old (Figure 1F). The average kainate-induced gamma power in slices of the aged mice was decreased compared with that in the 3-month-old PS mice (Figure 1G, *p* < 0.01 at CA1, *p* < 0.05 at CA3 and DG, *n* = 8–9). A previous study showed that aged mice exhibit synaptic alterations, such as changes in spine density and ion channel function, and our data provide evidence for further neurofunctional changes in the aged mice, which are associated with cognitive impairment (Tombaugh et al., 2005; Benito et al., 2015). These results demonstrated that a deficit in gamma oscillations was observed in both PSAPP mice and aged PS mice.

### Ameliorating Effect of an Histone Deacetylase Inhibitor on the Gamma Oscillation Deficit in PSAPP Mice

**FIGURE 2:**
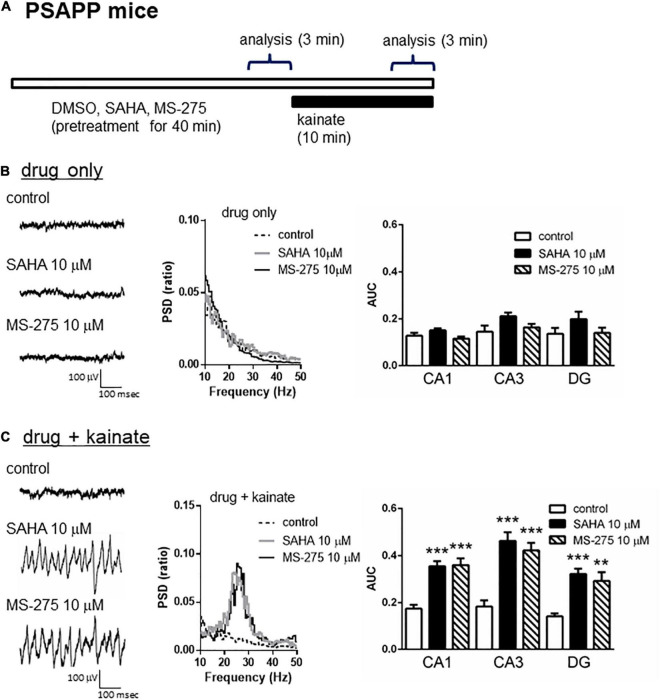
HDAC inhibitors rescued the deficits in the kainate-induced gamma oscillations in the CA1, CA3, and DG in slices prepared from PSAPP mice. **(A)** A schematic for drug application and analysis. **(B)** Representative traces (left panel) and PSD (middle panel) at CA3 prior to kainate application, indicating that the local field potentials were not changed in slices treated *in vitro* with DMSO 0.1% (indicated as control), SAHA (10 μM) or MS-275 (10 μM) for 40 min. The average gamma power was quantified (right panel) and was not changed by SAHA or MS-275 (*p* > 0.05, control; *n* = 6, SAHA; *n* = 6, MS-275; *n* = 7). **(C)** Representative traces (left panel) and PSD (middle panel) at CA3 after kainate application, indicating that SAHA (10 μM) and MS-275 (10 μM) strongly rescued the deficit in the kainate-induced gamma oscillations in slices from PSAPP mice. The average power of the kainate-induced gamma oscillations was significantly increased by SAHA and MS-275 (SAHA; *p* < 0.001 at CA1, CA3, and DG, MS-275; *p* < 0.001 at CA1 and CA3, *p* < 0.01 at DG, control; *n* = 6, SAHA; *n* = 6, MS-275; *n* = 7). **p < 0.01 and ***p < 0.001.

**FIGURE 3:**
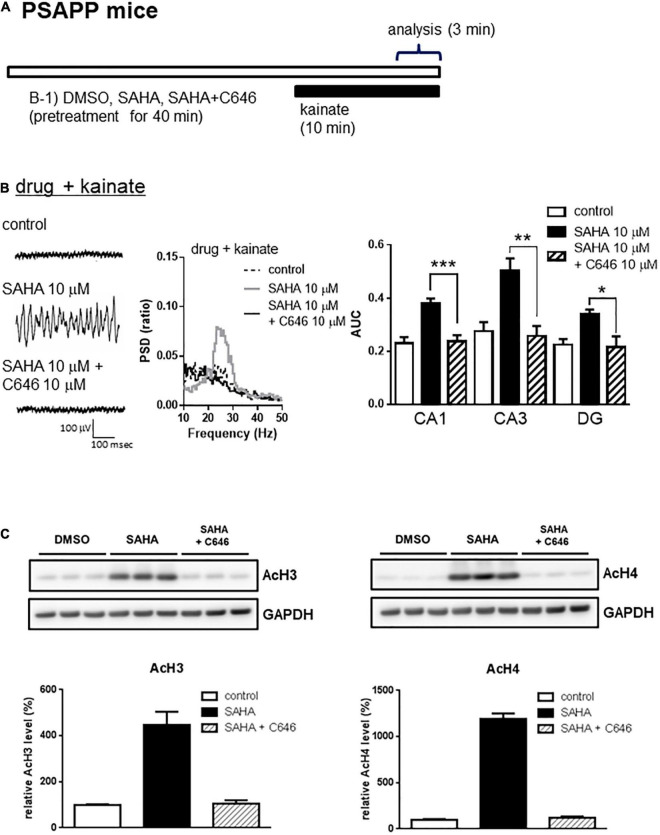
The HAT inhibitor abolished the effect of the HDAC inhibitor on the deficits in gamma oscillations in the CA1, CA3, and DG in slices prepared from PSAPP mice. **(A)** A schematic for drug application and analysis. **(B)** Representative traces (left panel) and PSD (middle panel) at CA3, indicating that SAHA (10 μM) strongly rescued the deficit in kainate-induced gamma oscillations in slices from PSAPP mice, which were abolished in slices treated with SAHA (10 μM) and C646 (10 μM). The average power of kainate-induced gamma oscillations in slices treated with SAHA and C646 was significantly decreased compared to that of SAHA only-treated slices (*p* < 0.001 at CA1, *p* < 0.01 at CA3, *p* < 0.05 at DG, control; *n* = 6, SAHA; *n* = 8, SAHA with C646; *n* = 7). **(C)** Acetylation of histone H3 and H4 by acute *in vitro* treatment with the HDAC inhibitor SAHA and competition with the HAT inhibitor C646. On the upper panel, immunoblotting of hippocampal protein extracts from PSAPP slices treated *in vitro* with SAHA (10 μM) alone and in combination with C646 (10 μM). On the bottom panel, the averages of the relative levels of both acetylated H3 and acetylated H4 were increased by SAHA but were abolished by co-treatment with C646 (control; *n* = 3, SAHA; *n* = 3, SAHA with C646; *n* = 3). *p < 0.05, **p < 0.01, ***p < 0.001.

A previous study has demonstrated that acute *in vitro* treatment with an HDAC inhibitor for 40 min enhanced the induction of long-term potentiation at Schaffer-collateral synapses in the CA1 of the hippocampus (Alarcón et al., 2004). Using this drug treatment protocol, we investigated whether acute *in vitro* application of the HDAC inhibitors SAHA and MS-275 could rescue deficits in gamma oscillations in PSAPP mice. The application of neither SAHA (10 μM) nor MS-275 (10 μM) alone for 40 min (Figure 2A) affected the oscillatory activity of local field potentials (Figure 2B left and middle panel), and the average gamma power was not changed (Figure 2B right panel, *p* > 0.05, *n* = 6–7). In contrast, both drugs rescued the deficits in kainate-induced gamma oscillations in PSAPP mice (Figure 2C left and middle panel). The average power of the kainate-induced gamma oscillations was significantly increased by SAHA and MS-275 (Figure 2C right panel, SAHA; *p* < 0.001 at CA1, CA3 and DG, MS-275; *p* < 0.001 at CA1 and CA3, *p* < 0.01 at DG, *n* = 6–7). To investigate whether this ameliorating effect of the HDAC inhibitor SAHA was mediated by histone acetylation, we evaluated the effect of the HAT inhibitor C646 on the effect of SAHA. As shown in Figures 3A,B, the ameliorating effect of SAHA (10 μM) on deficits in kainate-induced gamma oscillations in PSAPP mice was abolished by the co-application of C646 (10 μM). The average kainate-induced gamma power in slices treated with SAHA alone was significantly different from that of those treated with SAHA and C646 (Figure 3B right panel, *p* < 0.001 at CA1, *p* < 0.01 at CA3, *p* < 0.05 at DG, *n* = 7–8). To confirm that acute *in vitro* treatment with SAHA increased histone acetylation levels in hippocampal slices, we measured the levels of acetylated histone H3 and H4 in PSAPP slices by immunoblotting. Treatment with SAHA (10 μM) for 40 min greatly increased the acetylation of histones H3 and H4, and the effect was abolished in slices treated with SAHA and C646 (Figure 3C, *n* = 3). These results showed that HDAC inhibition and subsequent elevation of histone acetylation rescued deficits in gamma oscillations in PSAPP mice.

### Ameliorating Effect of Nuclear Receptor Family 4a Activation on the Gamma Oscillation Deficit in PSAPP Mice

**FIGURE 4:**
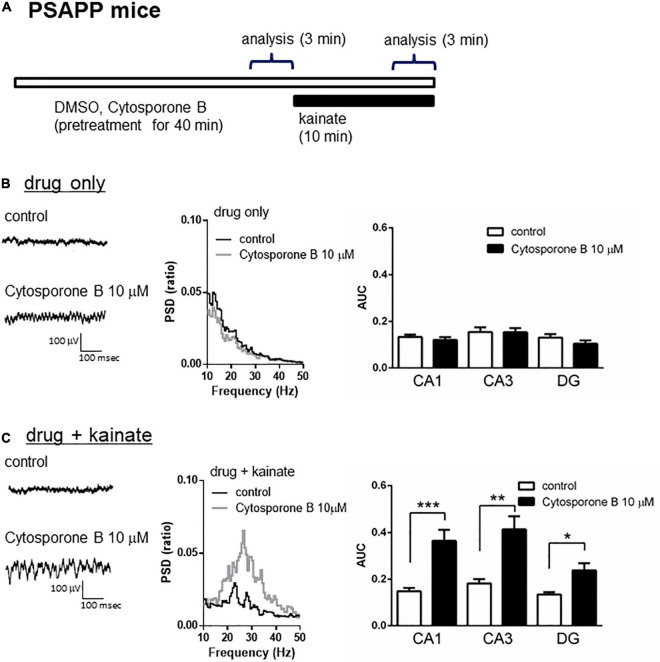
The NR4a agonist rescued the deficits in kainate-induced gamma oscillations in the CA1, CA3 and DG in slices prepared from PSAPP mice. **(A)** A schematic for drug application and analysis. **(B)** Representative traces (left panel) and PSD (middle panel) at CA3 prior to kainate application, indicating that the local field potentials were not changed in slices treated *in vitro* with DMSO 0.1% (indicated as control) and cytosporone B (10 μM) for 40 min. The average gamma power was quantified (right panel) and was not changed by cytosporone B (*p* > 0.05, control; *n* = 7, cytosporone B; *n* = 7). **(C)** Representative traces (left panel) and PSD (middle panel) at CA3 after kainate application, indicating that cytosporone B (10 μM) strongly rescued deficit in the kainate-induced gamma oscillations in slices from PSAPP mice. The average power of kainate-induced gamma oscillations was significantly increased by cytosporone B (*p* < 0.001 at CA1, *p* < 0.01 at CA3, *p* < 0.05 at DG, control; *n* = 7, cytosporone B; *n* = 7). *p < 0.05, **p < 0.01, ***p < 0.001.

A previous study proposed that activation of the nuclear receptor family 4a (NR4a) receptor is one of the epigenetic modification related to HDAC inhibition and the improvement of cognitive functions. HDAC inhibition elevated histone acetylation and caused the activation of its downstream NR4a receptor, which subsequently enhanced memory formation (Vecsey et al., 2007; Hawk et al., 2012). Given that HDAC inhibition rescued the gamma oscillation deficit in PSAPP mice, we investigated the effect of NR4a receptor activation as one of the epigenetic modification related to HDAC inhibition. Acute application of the NR4a receptor agonist cytosporone B (10 μM) for 40 min (Figure 4A) did not affect the gamma oscillatory activity in PSAPP mice (Figure 4B left and middle panel). The average gamma power was not significantly changed by cytosporone B (Figure 4B right panel, *p* > 0.05, *n* = 7). In contrast, cytosporone B rescued the deficit in kainate-induced gamma oscillatory activity in PSAPP mice (Figure 4C left and middle panel). The average kainate-induced gamma power was significantly increased by cytosporone B (Figure 4C right panel, *p* < 0.001 at CA1, *p* < 0.01 at CA3, *p* < 0.05 at DG, *n* = 7). These results demonstrated that NR4a activation as one of the epigenetic modification related to HDAC inhibition rescued deficits in kainate-induced gamma oscillations in PSAPP mice.

### Regulation of Gamma Oscillations by Histone Acetylation in the CA1, CA3, and DG of Hippocampal Slices From Wild-Type Mice

**FIGURE 5:**
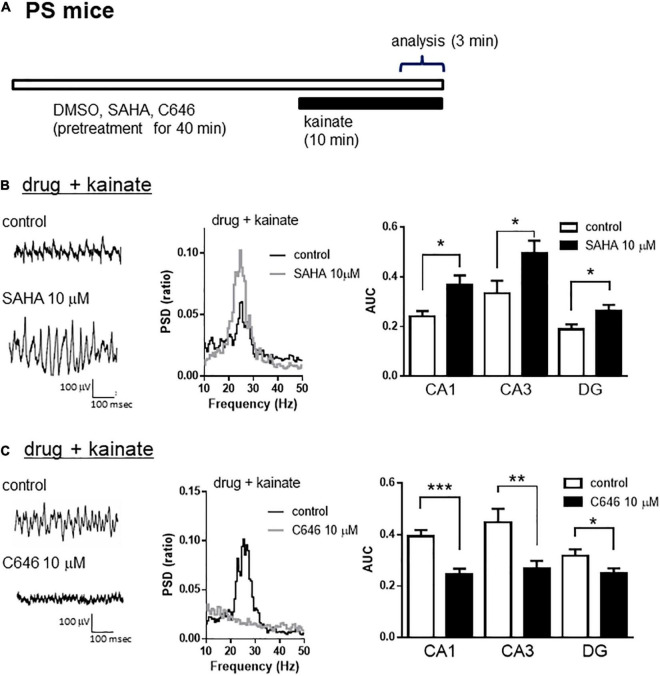
The HDAC inhibitor increased, while the HAT inhibitor decreased the kainate-induced gamma oscillations in the CA1, CA3, and DG of hippocampal slices prepared from wild-type mice. **(A)** A schematic for the drug application and analysis. **(B)** Representative traces (left panel) and PSD (middle panel) at CA3, indicating that 100 nM of kainate (indicated as control) induced gamma oscillations in slices from wild-type mice, an effect that was further augmented by pretreatment with SAHA (10 μM). The average power of the kainate-induced gamma oscillations (right panel) was significantly increased by SAHA (*p* < 0.05 at CA1, CA3, and DG, control; *n* = 7, SAHA; *n* = 7). **(C)** Representative traces (left panel) and PSD (middle panel) at CA3, indicating that 200 nM of kainate (indicated as control) induced gamma oscillations in slices from wild-type mice that were abolished by pretreatment with C646 (10 μM). The average power of kainate-induced gamma oscillations (right panel) was significantly decreased by C646 (*p* < 0.001 at CA1, *p* < 0.01 at CA3, *p* < 0.05 at DG, control; *n* = 7, C646; *n* = 8). *p < 0.05, **p < 0.01, ***p < 0.001.

A previous study demonstrated that the inhibition of HDAC increased histone acetylation and enhanced synaptic functions such as spine density and/or long-term potentiation in wild-type mice, which was associated with memory enhancement in maze or fear conditioning tests (Guan et al., 2009; Gräff and Tsai, 2013). We investigated whether histone acetylation could modulate kainate-induced gamma oscillations in hippocampal slices from wild-type mice. Using a lower concentration of kainate (100 nM), we detected that the acute application of SAHA (10 μM) for 40 min (Figure 5A) enhanced kainate-induced gamma oscillatory activity in the hippocampus of wild-type mice (Figure 5B left and middle panel). The average gamma power was significantly increased by SAHA (Figure 5B right panel, *p* < 0.05 at CA1, CA3, and DG, *n* = 7). Conversely, acute application of C646 (10 μM) for 40 min (Figure 5A) abolished the gamma oscillatory activity induced by kainate (200 nM) in wild-type mice (Figure 5C left and middle panel). The average gamma power was significantly decreased by C646 (Figure 5C right panel, *p* < 0.001 at CA1, *p* < 0.01 at CA3, *p* < 0.05 at DG, *n* = 7–8). Together with the effect of SAHA in PSAPP mice, these results indicate that histone acetylation levels regulate kainate-induced gamma oscillations in the hippocampus of both wild-type and AD model mice.

### Impairments in the Intrinsic Excitability of Fast Spiking Interneurons in the Hippocampus of PSAPP Mice and Their Rescue by an Histone Deacetylase Inhibitor

**FIGURE 6:**
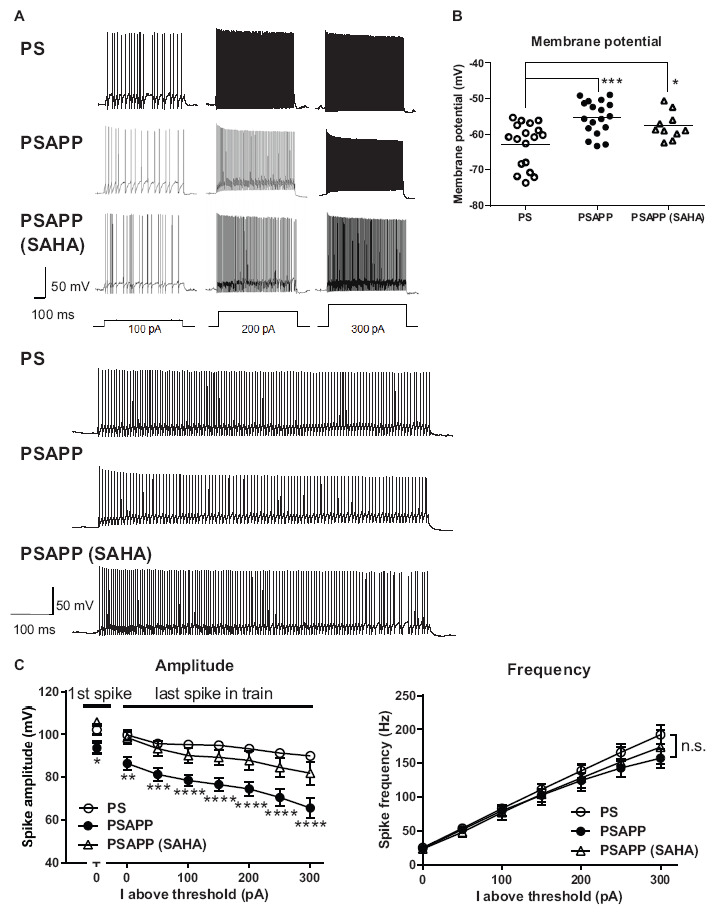
Dysfunction of the intrinsic excitability of fast spiking interneurons in the hippocampus of PSAPP mice and its rescue by the HDAC inhibitor SAHA. **(A)** Representative traces indicating that the amplitude of action potentials induced by current steps of increasing amplitude (100 pA to 300 pA, and magnified traces at 300 pA) into visually identified hippocampal interneurons at the CA1 stratum pyramidal-oriens border was smaller in PSAPP mice than PS mice, which was rescued by SAHA (10 μM). **(B)** Fast spiking interneurons in PSAPP mice had more depolarized resting membrane potentials than those in PS mice, which was not rescued by SAHA (PS mice; *n* = 18 vs PSAPP mice; *n* = 18, *p* < 0.001, PSAPP mice treated with SAHA; *n* = 10, *p* < 0.05). **(C)** The averaged amplitude of the action potentials induced by each current injection was significantly smaller in PSAPP mice than PS mice, which was rescued by SAHA (PS mice; *n* = 18 vs PSAPP mice; *n* = 18, PSAPP mice treated with SAHA; *n* = 10, *p < 0.05, **p < 0.01, ***p < 0.001, ****p < 0.0001). There was no difference in the firing frequency of interneurons among hippocampal slices of PSAPP mice, those of PS mice and those of PSAPP mice treated with SAHA (PS mice; *n* = 18 vs PSAPP mice; *n* = 18, *p* > 0.05, PSAPP mice treated with SAHA; *n* = 10, *p* > 0.05).

To investigate the mechanisms underlying the deficit in gamma oscillations in PSAPP mice and its rescue following treatment with SAHA, we evaluated the function of fast spiking interneurons, which were reported to mainly regulate the gamma oscillation (Bartos et al., 2007; Cardin et al., 2009). Previous studies have demonstrated that dysfunction of fast spiking interneurons in the hippocampus and neocortex was implicated in psychologic disorders including AD (Lewis et al., 2012; Marin, 2012; Verret et al., 2012; Hazra et al., 2013) and was related to a reduction in gamma oscillations accompanied by cognitive impairment (Verret et al., 2012; Etter et al., 2019; Chung et al., 2020). First, we performed whole-cell patch clamp recording from visually identified fast spiking interneurons at the CA1 stratum pyramidal-oriens border and examined their resting membrane potentials and action potential properties using current steps of increasing amplitude (800 ms, 50 pA steps). In the assessment of resting membrane potentials, fast spiking interneurons in PSAPP mice had a more depolarized resting membrane potentials than that in PS mice (Figure 6A, PSAPP mice: −55.29 ± 1.10 mV, *n* = 18 vs PS mice: −62.80 ± 1.46 mV, *n* = 18, *p* < 0.001). In the assessment of the action potential properties, the amplitude of the action potential induced by each current injection was significantly smaller in PSAPP mice than that in PS mice (Figures 6A,C left panel). By contrast, there was no difference in the firing frequency of interneurons (Figure 6C right panel). Acute treatment with SAHA (10 μM) in slices from PSAPP mice for 50–90 min did not change either the depolarized resting membrane potentials or the current injection-induced firing frequency of fast spiking interneurons in PSAPP mice (Figures 6B,C right panel). By contrast, SAHA restored the amplitude of the action potentials in fast spiking interneurons of PSAPP mice (Figure 6C left panel). Thus, PSAPP mice had a dysfunction of fast spiking interneurons of the hippocampus. The ameliorating effects of SAHA on gamma oscillation deficits could be attributed to be the rescue of the low intrinsic excitability of fast spiking interneurons by SAHA, a novel mechanism for the rescue of cognitive dysfunctions by SAHA.

### Increase of Spontaneous Activity and Decrease of Kainate-Induced Activity of Fast Spiking Interneurons in the Hippocampus of PSAPP Mice and Its Rescue by Histone Deacetylase Inhibitor

**FIGURE 7:**
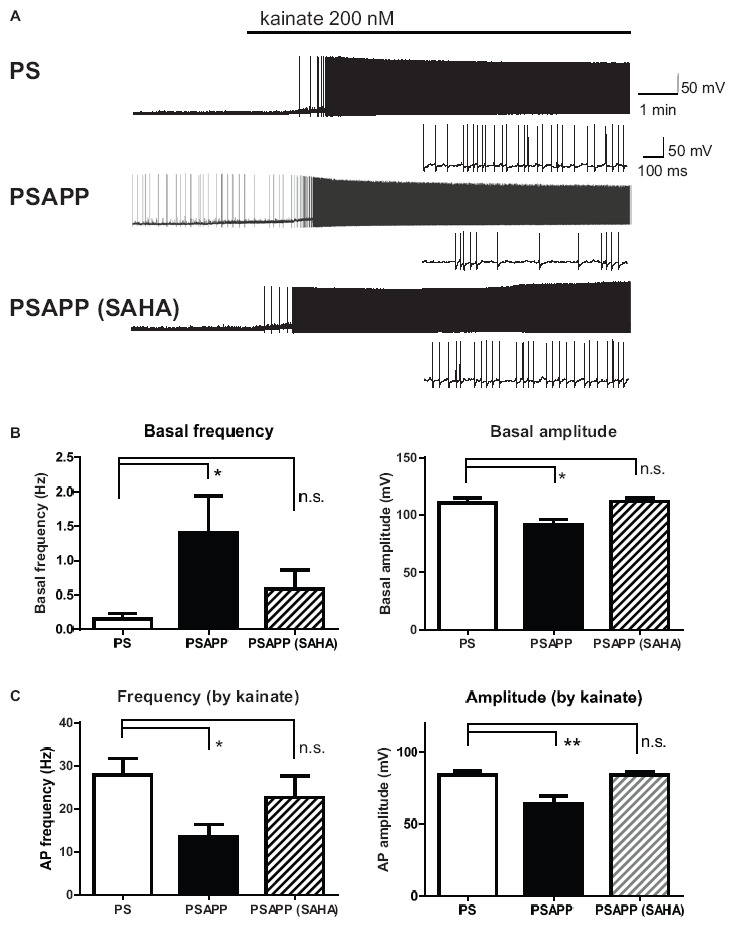
Increase of basal spontaneous firing activity and decrease of kainate-induced activity of fast spiking interneurons in PSAPP mice and their rescue by the HDAC inhibitor SAHA. **(A)** Representative traces indicating increased spontaneous firing activity prior to kainate application and deficits in the kainate-induced activity of fast spiking interneurons in PSAPP mice (total and magnified traces during kainate application), both of which were rescued by the HDAC inhibitor SAHA (10 μM). **(B)** Prior to kainate application, fast spiking interneurons in PSAPP mice more frequently elicited spontaneous action potentials than those in PS mice (PSAPP mice: *n* = 18 vs PS mice: *n* = 18, *p* < 0.05) and their amplitudes in PSAPP mice were smaller than those in PS mice (PSAPP mice: *n* = 13 vs PS mice: *n* = 9, *p* < 0.05). This altered spontaneous firing activity in PSAPP mice was returned to that in the PS mice by SAHA treatment, regarding both amplitude (PSAPP mice treated with SAHA: *n* = 5 vs PS mice: *n* = 9, *p* > 0.05) and frequency (PSAPP mice treated with SAHA: *n* = 10 vs PS mice: *n* = 18, *p* > 0.05). **(C)** Kainate-induced firing frequency and amplitude in PSAPP mice were significantly smaller than those in PS mice (frequency: PSAPP mice, *n* = 18 vs PS mice, *n* = 18, *p* < 0.05; amplitude: PSAPP mice, *n* = 18 vs PS mice, *n* = 18, *p* < 0.01). This decreased kainate-induced activity in PSAPP mice was returned to that in PS mice by SAHA treatment, regarding both frequency (PSAPP mice treated with SAHA: *n* = 10 vs PS mice: *n* = 18, *p* > 0.05) and amplitude (PSAPP mice treated with SAHA: *n* = 10 vs PS mice: *n* = 18, *p* > 0.05). *p < 0.05 and **p < 0.01.

Next, we investigated spontaneous and kainate-induced action potentials of fast spiking interneurons in PSAPP mice. A previous study demonstrated that kainate at a concentration of 200 nM increased the frequency of spike firing in interneurons in hippocampal slices, whose increased single neuronal firing activity is needed for the development of a gamma oscillation (Fisahn et al., 2004; Andersson et al., 2012). Moreover, as shown in Supplementary Figure 1, we have demonstrated that the kainate-induced firing activity of fast spiking interneurons was not abolished by AMPA receptor antagonist GYKI53655 (12.5 μM) and GABA_A_ receptor antagonist bicuculline (10 μM) while it was completely abolished by kainate/AMPA receptor antagonist CNQX (10 μM). This result is supported by previous study (Fisahn et al., 2004; Bartos et al., 2007; Andersson et al., 2012) indicating that kainate directly acts on kainate receptors in fast spiking interneurons and contributes to generate gamma oscillation. In the present study, we measured the frequency and amplitude of action potentials of fast spiking interneurons following treatment of hippocampal slices of PSAPP mice with kainate. Before treatment with kainate, fast spiking interneurons in PSAPP mice elicited spontaneous action potentials more frequently than those in PS mice (Figures 7A,B left panel, PSAPP mice: 1.41 ± 0.53 Hz, *n* = 18 vs PS mice: 0.15 ± 0.08 Hz, *n* = 18, *p* < 0.05) and their amplitudes in PSAPP mice were smaller than those in PS mice (Figures 7A,B right panel, PSAPP mice: 91.49 ± 4.85 mV, *n* = 13 vs PS mice: 110.34 ± 4.46 mV, *n* = 9, *p* < 0.05). After application of kainate (200 nM), fast spiking interneurons increased firing activity in both PSAPP and PS mice, but these kainate-induced changes in firing frequency and amplitude in PSAPP mice were significantly smaller than those in PS mice (Figure 7C left panel, frequency: 13.52 ± 2.85 Hz in PSAPP mice, *n* = 18 vs 27.85 ± 3.89 Hz in PS mice, *n* = 18, *p* < 0.05; Figure 7C right panel, amplitude: 64.40 ± 5.32 mV in PSAPP mice, *n* = 18 vs 84.25 ± 2.75 mV in PS mice, *n* = 18, *p* < 0.01). Acute treatment with SAHA (10 μM) in PSAPP mice rescued the increased spontaneous firing activity without kainate and the decreased kainate-induced firing activity (Figures 7A–C). In summary, following treatment with SAHA, spontaneous firing activity and kainate-induced firing activity of fast spiking interneurons in PSAPP mice were returned to that observed in PS mice (Figures 7B,C). The rescue of gamma frequency-specific firing activity of fast spiking interneurons by SAHA had similar time course with generation of gamma oscillation (Supplementary Figure 2). Besides, the average peak frequency of kainate-induced action potentials increased by SAHA (22.6 ± 4.93 Hz, *n* = 10) shows close to the peak frequency of the kainate-induced gamma oscillations increased by SAHA (24.5 ± 1.15 Hz, CA1 area, *n* = 6). These results suggest that HDAC inhibition rescues activity of fast spiking interneuron both before and after kainate application, accompanied with improvement of decrease in gamma oscillations in the hippocampus of PSAPP mice.

### Ameliorating the Effect of Donepezil on the Gamma Oscillation Deficits in PSAPP Mice

**FIGURE 8:**
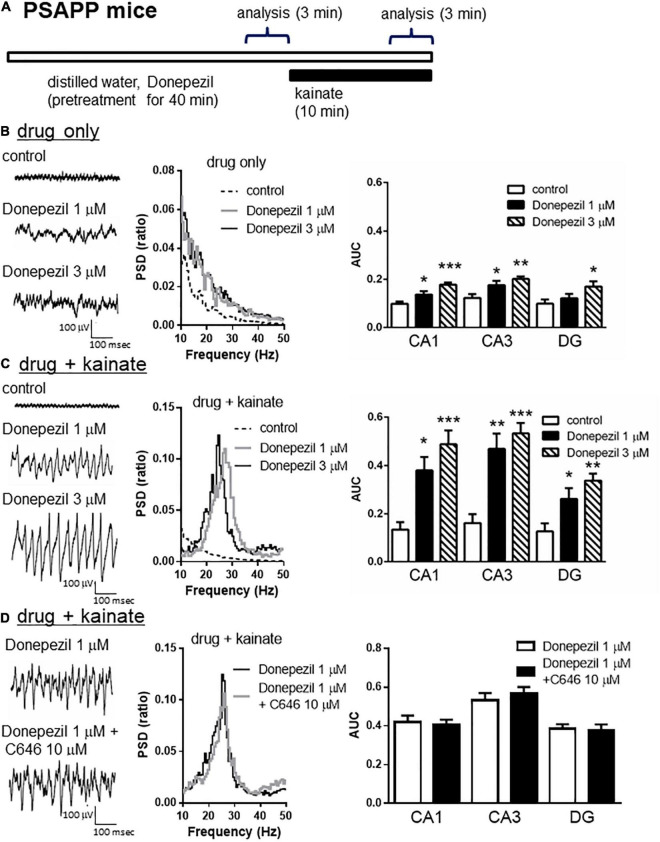
Donepezil rescued the deficits in kainate-induced gamma oscillations in the CA1, CA3, and DG in slices prepared from PSAPP mice, which is independent of histone acetylation. **(A)** A schematic for the drug application and analysis. **(B)** Representative traces (left panel) and PSD (middle panel) at CA3 prior to kainate application, indicating that the local field potentials were slightly increased in slices of PSAPP mice treated *in vitro* with donepezil (1, 3 μM) for 40 min. The average gamma power of CA1, CA3, and DG were quantified (right panel) and were slightly increased by donepezil (1 μM; *p* < 0.05 at CA1 and CA3, 3 μM; *p* < 0.001 at CA1, *p* < 0.01 at CA3, *p* < 0.05 at DG, control; *n* = 5, 1 μM; *n* = 5, 3 μM; *n* = 6). **(C)** Representative traces (left panel) and PSD (middle panel) at CA3 after kainate application, indicating that donepezil rescued the deficit in kainate-induced gamma oscillation in slices from PSAPP mice. The average power of kainate-induced gamma oscillation (right panel) was significantly increased by donepezil (1 μM; *p* < 0.01 at CA3, *p* < 0.05 at CA1 and DG, 3 μM; *p* < 0.001 at CA1 and CA3, *p* < 0.01 at DG, control; *n* = 5, 1 μM; *n* = 5, 3 μM; *n* = 6). **(D)** Representative traces (left panel) and PSD (middle panel) at CA3 indicating that donepezil (1 μM) strongly rescued the deficit in kainate-induced gamma oscillations in slices from PSAPP mice, which was not abolished in slices treated with donepezil and C646 (10 μM). The average power of kainate-induced gamma oscillation was not significantly different between donepezil alone and co-treatment with C646 (*p* > 0.05, donepezil; *n* = 7, donepezil with C646; *n* = 8). *p < 0.05, **p < 0.01, ***p < 0.001.

To validate the gamma oscillation deficit as a feature of cognitive impairment, we investigated whether deficits in gamma oscillation in PSAPP mice could be rescued by the AChE inhibitor donepezil, which has been approved for the palliative treatment of cognitive impairment in AD patients (Whitehead et al., 2004; Birks and Harvey, 2018). As shown in Figures 8A,B, treatment with donepezil for 40 min at concentrations of 1 and 3 μM alone slightly increased neuronal activity, as indicated by a significant increase in the average gamma power (Figure 8B right panel, 1 μM; *p* < 0.05 at CA1 and CA3, 3 μM; *p* < 0.001 at CA1, *p* < 0.01 at CA3, *p* < 0.05 at DG, *n* = 5–6). This is different from the effects of SAHA on the deficits in gamma oscillations via histone acetylation. Furthermore, donepezil (1 and 3 μM) rescued deficits in kainate-induced gamma oscillations in PSAPP mice (Figure 8C left and middle panel). The average kainate-induced gamma power was significantly increased by donepezil (Figure 8C right panel, 1 μM; *p* < 0.01 at CA3, *p* < 0.05 at CA1 and DG, 3 μM; *p* < 0.001 at CA1 and CA3, *p* < 0.01 at DG, *n* = 5–6). These results are consistent with previous reports that AChE inhibitors increase gamma oscillatory activity *in vivo* (Joosen and van Helden, 2007; Phillips et al., 2007). Moreover, the present study demonstrated that donepezil rescued the deficit in kainate-induced gamma oscillation in PSAPP mice. Next, to investigate whether the ameliorating effects of donepezil on the deficits in gamma oscillations in PSAPP mice are involved in histone acetylation, we evaluated the effect of C646 on the ameliorating effect of donepezil. We found that C646 (10 μM) did not alter the effect of donepezil (1 μM) on the gamma oscillation deficit in PSAPP mice (Figure 8D left and middle panel). The average kainate-induced gamma power in slices treated with donepezil alone was not significantly different from those treated with both donepezil and C646 (Figure 8D right panel, *p* > 0.05, *n* = 7–8). This result indicated that the effects of donepezil did not require histone acetylation.

### Donepezil Partially Restored Spontaneous Activity but Not Kainate-Induced Firing Activity of Fast Spiking Interneuron Activity

**FIGURE 9:**
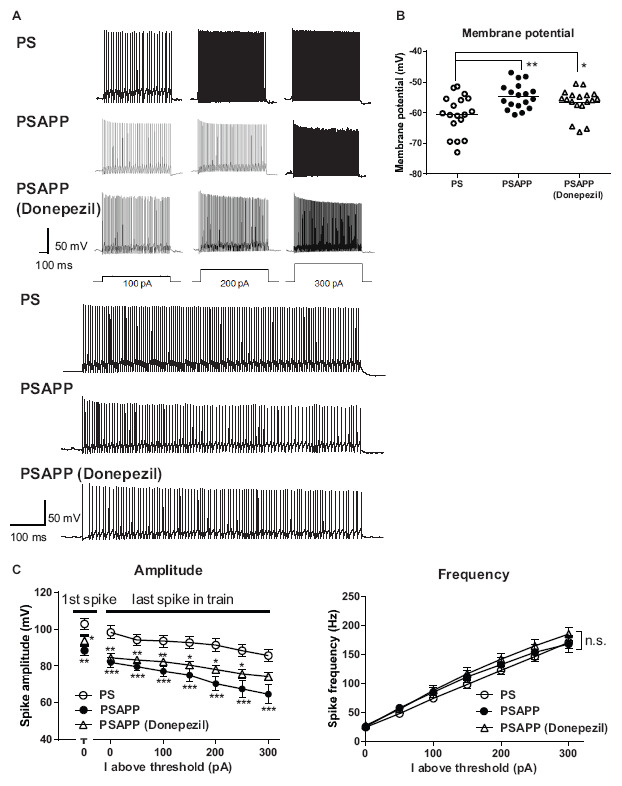
Donepezil did not rescue the depolarized resting membrane potentials and changes of intrinsic excitability of fast spiking interneurons in the hippocampus of PSAPP mice. **(A)** Representative traces, indicating that the amplitude of the action potentials induced by current steps of increasing amplitude (100 pA to 300 pA, and magnified traces at 300 pA) into visually identified hippocampal interneurons at the CA1 stratum pyramidal-oriens border was smaller in PSAPP mice than that in PS mice, which was not rescued by donepezil (1 μM). **(B)** Fast spiking interneurons in PSAPP mice had more depolarized resting membrane potentials than that in PS mice, which were not rescued by SAHA (PS mice; *n* = 18 vs PSAPP mice; *n* = 18, *p* < 0.01, PSAPP mice treated with donepezil; *n* = 18, *p* < 0.05). **(C)** Averaged amplitude of action potential induced by each current injection was significantly decreased in PSAPP mice as compared to PS mice, which was not rescued by donepezil (PS mice; *n* = 18 vs PSAPP mice; *n* = 18, PSAPP mice treated with donepezil; *n* = 18, *p < 0.05, **p < 0.01, ***p < 0.001). There was no difference in the firing frequency of interneurons among hippocampal slices of PSAPP mice, those of PS mice and those of PSAPP mice treated with donepezil (PS mice; *n* = 18 vs PSAPP mice; *n* = 18, *p* > 0.05, PSAPP mice treated with donepezil; *n* = 18, *p* > 0.05).

**FIGURE 10:**
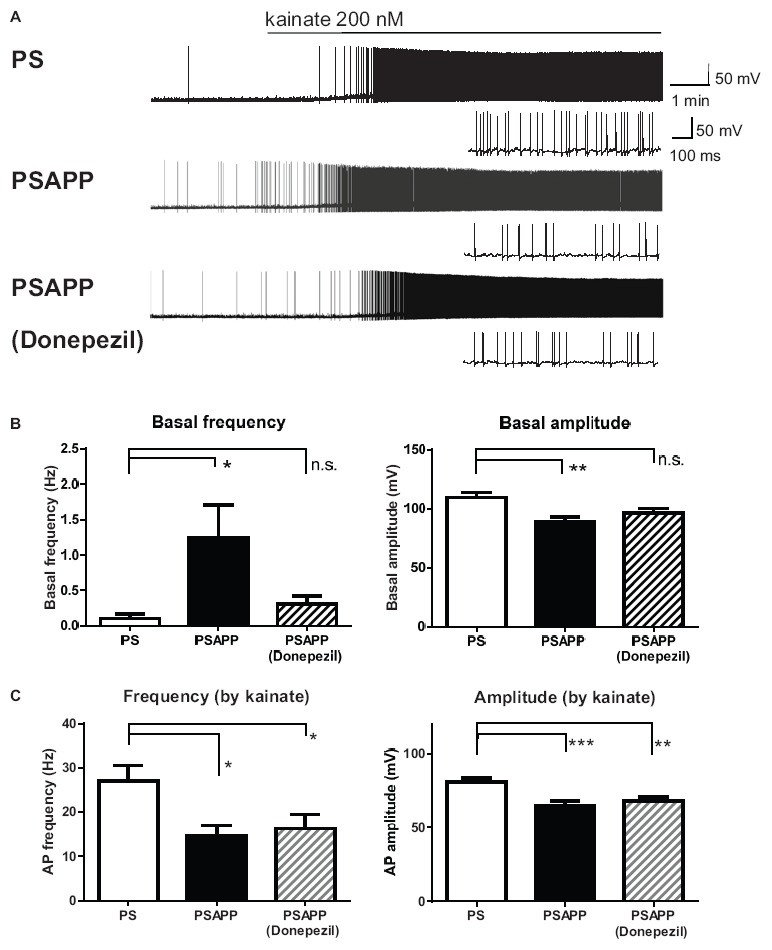
Donepezil rescued only the increased spontaneous firing activity but not the reduction kainate-induced activity of fast spiking interneurons in PSAPP mice. **(A)** Representative traces indicating increased spontaneous firing activity prior to kainate application, and reduction in kainate-induced activity of fast spiking interneurons in PSAPP mice (total and magnified traces during kainate application). Only the increased spontaneous firing activity was rescued by donepezil (1 μM). **(B)** Prior to kainate application, fast spiking interneurons in PSAPP mice more frequently elicited spontaneous action potentials than those in PS mice (PSAPP mice: *n* = 18 vs PS mice: *n* = 18, *p* < 0.05) and their amplitudes in PSAPP mice were smaller than those in PS mice (PSAPP mice: *n* = 14 vs PS mice: *n* = 11, *p* < 0.01). This altered spontaneous firing activity in PSAPP mice was returned to that in PS mice by donepezil treatment, regarding both amplitude (PSAPP mice treated with donepezil: *n* = 12 vs PS mice: *n* = 11, *p* > 0.05) and frequency (PSAPP mice treated with donepezil: *n* = 18 vs PS mice: *n* = 18, *p* > 0.05). **(C)** Kainate-induced firing frequency and amplitude in PSAPP mice were significantly smaller than those in PS mice (frequency: PSAPP mice, *n* = 18 vs PS mice, *n* = 18, *p* < 0.05, amplitude: PSAPP mice, *n* = 18 vs PS mice, *n* = 18, *p* < 0.001). This altered kainate-induced activity in PSAPP mice was not returned to that in PS mice by donepezil treatment, regarding both frequency (PSAPP mice treated with donepezil: *n* = 18 vs PS mice: *n* = 18, *p* < 0.05) and amplitude (PSAPP mice treated with donepezil: *n* = 18 vs PS mice: *n* = 18, *p* < 0.01). *p < 0.05, **p < 0.01, ***p < 0.001.

To investigate whether the rescue of the gamma oscillations by donepezil is attributed to the restoration of the activity of fast spiking interneurons, we evaluated the effects of donepezil on the activity of fast spiking interneurons. Treatment with donepezil (1 μM) did not reverse the depolarized shift in the resting membrane potential in PSAPP mice (Figure 9B, PS mice: −60.61 ± 1.50 mV, *n* = 18 vs PSAPP mice treated with vehicle: −54.63 ± 0.97 mV, *n* = 18, *p* < 0.001 and PSAPP mice treated with donepezil: −56.59 ± 1.05 mV, *n* = 18, *p* < 0.05). Additionally, the reduced amplitude of the action potential induced by current injection in fast spiking interneurons from PSAPP mice was not rescued by donepezil (Figures 9A,C). Conversely, donepezil reversed the alteration in the spontaneous activity, regarding both firing frequency (Figures 10A,B left panel, PS mice: 0.10 ± 0.06 Hz, *n* = 18 vs PSAPP mice treated with vehicle: 1.25 ± 0.47 Hz, *n* = 18, *p* < 0.001 and PSAPP mice treated with donepezil: 0.30 ± 0.11 mV, *n* = 18, *p* > 0.05) and amplitude (Figures 10A,B right panel, PS mice: 109.37 ± 4.31 mV, *n* = 11 vs PSAPP mice treated with vehicle: 88.66 ± 4.17 mV, *n* = 14, *p* < 0.001 and PSAPP mice treated with donepezil: 96.46 ± 3.62 mV, *n* = 12, *p* > 0.05). Nevertheless, donepezil did not rescue the reduced kainate-induced firing activity of fast spiking interneurons in PSAPP mice, in terms of both firing rate (Figures 10A,C left panel, PS mice: 27.08 ± 3.47 Hz, *n* = 18 vs PSAPP mice treated with vehicle: 14.52 ± 2.45 Hz, *n* = 18, *p* < 0.05 and PSAPP mice treated with donepezil: 16.32 ± 3.13 Hz, *n* = 18, *p* < 0.05) and amplitude (Figures 10A,C right panel, PS mice: 80.86 ± 2.71 mV, *n* = 18 vs PSAPP mice treated with vehicle: 64.77 ± 3.08 mV, *n* = 18, *p* < 0.001 and PSAPP mice treated with donepezil: 68.01 ± 2.83 mV, *n* = 18, *p* < 0.01). Overall, donepezil reversed the increased spontaneous firing activity in PSAPP mice without affecting the reduction of both the intrinsic excitability and the kainate-induced firing activity of fast spiking interneurons. The summary of present results was shown in the Table 1.

**TABLE 1:** Summary of deficits of functions of fast spiking interneurons in PSAPP mice and the ameliorating effects of SAHA and donepezil.

Parameter			Deficits in PSAPP mice	Effect of SAHA	Effect of donepezil
Basal state	Resting membrane potential		Depolarized shift	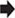	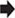
	Spontaneous activity	Amplitude	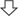	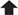	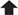
		Frequency	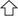	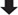	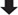
Activated state	Current injection-induced activity	Amplitude	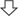	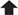	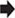
		Frequency	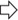	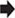	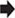
	Kainate-induced activity	Amplitude	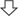	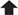	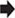
		Frequency	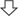	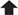	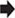

*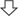, decrease vs PS 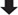, decrease vs PSAPP.*

*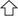, increase vs PS 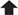, increase vs PSAPP.*

*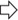, no change 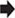, no change.*

### Excitatory Synaptic Input to Fast Spiking Interneuron in PSAPP Mice Was Reduced and This Reduction Was Rescued by Suberoylanilide Hydroxamic Acid and Donepezil

**FIGURE 11:**
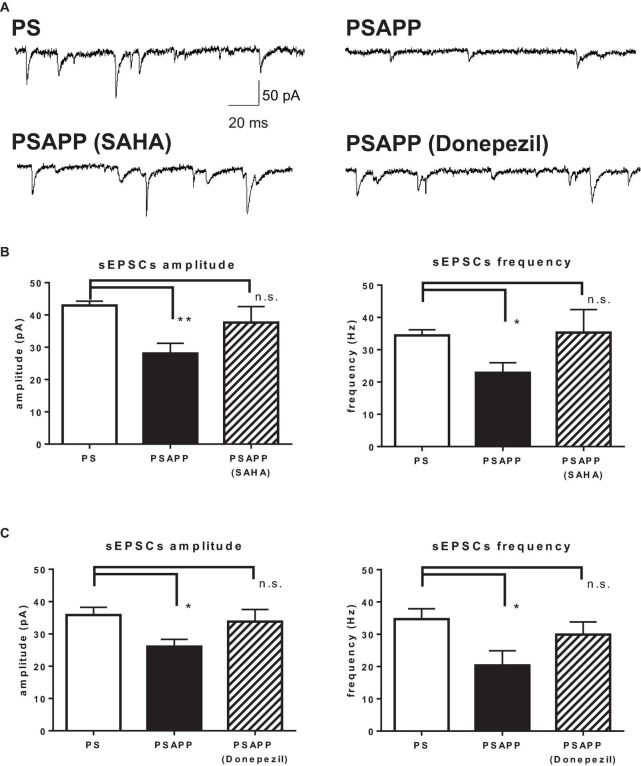
SAHA and Donepezil rescued spontaneous excitatory synaptic inputs to fast spiking interneurons in PSAPP mice. **(A)** Representative traces indicating spontaneous excitatory postsynaptic currents (sEPSCs) in the fast spiking interneuron from PSAPP mice. **(B)** Frequency and amplitude of sEPSCs in PSAPP mice were significantly smaller than those in PS mice (frequency: PSAPP mice, *n* = 17 vs PS mice, *n* = 13, *p* < 0.05, amplitude: PSAPP mice, *n* = 17 vs PS mice, *n* = 13, *p* < 0.01). This reduction of sEPSCs in fast spiking interneurons in PSAPP mice was returned to that in PS mice by SAHA treatment, regarding both frequency (PSAPP mice treated with SAHA: *n* = 10 vs PS mice: *n* = 17, *p* > 0.05) and amplitude (PSAPP mice treated with SAHA: *n* = 10 vs PS mice: *n* = 17, *p* > 0.05). **(C)** Frequency and amplitude of sEPSCs in PSAPP mice were significantly smaller than those in PS mice (frequency: PSAPP mice, *n* = 9 vs PS mice, *n* = 9, *p* < 0.05, amplitude: PSAPP mice, *n* = 9 vs PS mice, *n* = 9, *p* < 0.05). This reduction of sEPSCs in fast spiking interneurons in PSAPP mice was returned to that in PS mice by donepezil treatment, regarding both frequency (PSAPP mice treated with donepezil: *n* = 9 vs PS mice: *n* = 9, *p* > 0.05) and amplitude (PSAPP mice treated with SAHA: *n* = 9 vs PS mice: *n* = 9, *p* > 0.05). *p < 0.05 and **p < 0.01.

Finally, we investigated whether SAHA and donepezil could affect the excitatory inputs to fast spiking interneuron in PSAPP mice. Whole cell patch clamp recordings were performed from fast spiking interneurons in hippocampus and spontaneous excitatory postsynaptic currents (sEPSCs) were analyzed, indicated by representative trace of sEPSCs in Figure 11A. In PSAPP mice, frequency and amplitude of sEPSCs were significantly smaller than those in PS mice (Figure 11B, left panel, frequency: 34.45 ± 1.71 Hz in PSAPP mice, *n* = 17 vs 22.86 ± 3.11 Hz in PS mice, *n* = 13, *p* < 0.05; right panel, amplitude: 42.93 ± 1.34 pA in PSAPP mice, *n* = 17 vs 28.07 ± 3.13 pA in PS mice, *n* = 13, *p* < 0.01). Acute treatment with SAHA (10 μM) rescued both reduced frequency and amplitude of EPSCs in fast spiking interneurons in PSAPP mice (Figure 11B). Similar effects were observed by treatment of donepezil (Figure 11C). Besides, rheobase, as a threshold value of currents for action potentials, in PSAPP mice was significantly lower than that in PS mice, which were rescued by SAHA and donepezil (Supplementary Figures 3A,B). By contrast, subthreshold oscillatory membrane potentials of 20–40 Hz gamma frequency were not changed in PSAPP mice and SAHA and donepezil does not change them (PSAPP mice treated with vehicle; 0.30 ± 0.01 vs SAHA; 0.29 ± 0.03, PS mice treat with vehicle; 0.33 ± 0.01, PSAPP mice treated with vehicle; 0.30 ± 0.01 vs donepezil; 0.33 ± 0.01, PS mice treated with vehicle; 0.33 ± 0.01). This suggested that basal subthreshold oscillatory membrane potentials in fast spiking interneurons were not changed in PSAPP mice. Thus, these results indicate that spontaneous excitatory synaptic inputs into fast spiking interneurons are rescued by SAHA and donepezil, which could be related to increasement of GABAergic inhibition in basal state (discussed below).

## Discussion

### The Gamma Oscillation in PSAPP Mice Was Impaired, Accompanied With Alteration of Fast Spiking Interneurons Activity in Basal and Activated State

The present study demonstrated a decrease in gamma oscillations in PSAPP mice, accompanied with activity of fast spiking interneurons in the hippocampus in basal and activated state (“basal state” means condition without kainate and “activated state” means condition under stimulation such as kainate or current injection). From several lines of evidence, two main mechanisms for the generation of gamma oscillations have been proposed: the interneuron network gamma (ING) and pyramidal interneuron network gamma (PING) models (Bartos et al., 2007; Salkoff et al., 2015). Depending on the specific conditions, either the ING or PING models may be the prominent mechanism (Bartos et al., 2007; Buzsaki and Wang, 2012), but the accumulating evidence favors the PING model as the likely mechanism for the types of gamma oscillations that are altered in Alzheimer disease (Verret et al., 2012; Hazra et al., 2013; Salkoff et al., 2015). In this model, both synapse-dependent and synapse-independent properties of fast spiking interneurons are crucial for the gamma oscillation; the PING model-based mechanism for the generation of gamma oscillations suggests that (1) PV-containing fast spiking inhibitory interneurons fire following excitatory synaptic inputs from pyramidal neurons. (2) This fast spiking causes interneuron-triggered strong inhibition that silences the activity of a local population of asynchronously firing pyramidal neurons. (3) Following the decay of the inhibitory effect, the postsynaptic pyramidal cells fire in synchrony. If synaptic inhibition is rhythmic at a gamma frequency, then the pyramidal cell activity becomes rhythmic as well, generating a synchronous gamma oscillation in the normal network (Bartos et al., 2007; Buzsaki and Wang, 2012; Salkoff et al., 2015). However, in a pathological state, abnormal synaptic inputs to fast spiking interneurons and/or their altered intrinsic firing activity weaken the temporal precision of GABAergic synaptic inhibition, causing deficits in the gamma oscillation. The deficits in synaptic inputs could be attributed to changes in phasic excitatory synaptic inputs from pyramidal neurons via glutamatergic AMPA receptors and the inhibitory synaptic regulation via GABA_A_ receptors of inhibitory interneurons and pyramidal neurons (Kodama et al., 2011; Salkoff et al., 2015). By contrast, deficits in intrinsic activity could be attributed to alteration of the intrinsic properties of sustained high-frequency firing of a single fast spiking interneuron, which are regulated by voltage-gated Kv3.1 potassium channels and/or the activation and recovery of voltage-gated Nav1.1 sodium channels (Hu et al., 2014). Together with previous reports (Verheugen et al., 1999; Verret et al., 2012; Hazra et al., 2013), these single neuronal changes in basal and activated state could trigger alteration of fast axonal action potential propagation and/or temporal precision of GABAergic synaptic transmission, resulting in deficits in gamma oscillations. The HDAC inhibitor SAHA rescued alteration of fast spiking interneurons activity in basal and activated state in AD model mice while donepezil rescued only in basal state. The possible molecular mechanisms are discussed in the following section.

### Histone Deacetylase Inhibition Improved Impairment of Gamma Oscillation in PSAPP Mice by Restoring Alteration of Fast Spiking Interneurons Activity in Basal and Activated State

At first, activity of fast spiking interneurons in basal state, demonstrated by decrease of rheobase and sEPSCs and sequentially increase of firing activity, was rescued by SAHA. This mechanism for rescue in basal state could be mediated by normalization of excitatory and/or inhibitory synaptic inputs to fast spiking interneurons. For example, elevation of GABAergic synaptic function was reported to occur following HDAC inhibition, and this may be a key mechanism for the restoration of abnormal spontaneous firing activity of fast spiking interneurons. Authement et al. (2015) reported that acute *in vitro* HDAC inhibition rescued GABAergic synaptic abnormalities through the epigenetic modification of the postsynaptic scaffolding A-kinase anchoring protein 79/150 (AKAP79/150), which subsequently increased the expression of GABA_A_ receptors at the GABAergic synapse. Moreover, HDAC inhibition elevated expression of GAD65 in central GABAergic neurons (Zhang et al., 2011; Tao et al., 2015). It is generally acknowledged that GAD65 is essential to the intensification of synaptic activity and acts by reversibly binding to the membrane of synaptic GABA vesicles and maintaining intracellular and intercellular GABA homeostasis (Walls et al., 2011). Therefore, it is possible that HDAC inhibition rescued basal spontaneous activity of fast spiking interneurons by increasing GABAergic synaptic function through the elevation of GABA_A_ receptors and/or GAD65 expression. This increasement of GABAergic inhibition could explain why rescue of deficits of sEPSCs along with increase of amplitude and frequency did caused neither further depolarization nor increase of basal activity of fast spiking interneurons, according to previous study (Mitchell and Silver, 2003; Bartos et al., 2007; Kispersky et al., 2012). This increasement of GABAergic inhibition is also consistent with the increasement of rheobase in fast spiking interneurons by SAHA. More importantly, deficits of fast spiking interneurons in activated state, demonstrated by reduction of current injection-induced excitability and kainate-induced spike activity, was rescued by SAHA but not donepezil. As a key molecule for the rescue of activity of them by HDAC inhibition in activated state, activation of voltage-gated sodium channels could be involved (Uchida et al., 2010; Matsushita et al., 2013) due to the fact that a previous study showed that increased function of voltage-gated sodium channels rescued both the decrease in intrinsic excitability of fast spiking interneurons and impairments in gamma oscillations in the cortex of AD model mice (Verret et al., 2012). Previous study also demonstrated that activation of fast spiking interneurons using optogenetic technique at gamma frequency induces gamma oscillation (Cardin et al., 2009) and that fast spiking neurons are firing synchronously at gamma frequency during gamma oscillation (Andersson et al., 2012). This is consistent with our result that SAHA rescued deficit of amplitude and frequency of kainate-induced action potentials and amplitude of current injection-induced action potentials in PSAPP mice. The rescue of kainate-induced action potentials by SAHA had similar time course and frequency properties with rescue of gamma oscillation, indicating that gamma frequency activity of fast spiking interneurons might be important for gamma oscillation. In the assessment of resting membrane potentials, fast spiking interneurons in PSAPP mice had a more depolarized resting membrane potentials than that in PS mice. This mechanism for depolarization of resting membrane potentials in PSAPP mice was not fully clarified, but the reduction of Na/K pump (Na/K-ATPase), which is important for regulation of resting membrane potentials, is reported in AD model mice (Ohnishi et al., 2015; Petrushanko et al., 2016). Since SAHA had no effect on depolarized resting membrane potential, it is little likelihood that Na/K-ATPase might contribute to the effects of SAHA. Together with previous studies (Cardin et al., 2009; Verret et al., 2012), SAHA could increase the function of voltage-gated sodium channels or change the composition of their subunits, specifically the fast activation subtype crucial for action potential generation, and subsequently improve activity of fast spiking interneurons in activated state by restoring the single neuronal firing properties of fast spiking interneurons. Recently, fast spiking firing properties of interneurons have been reported to be essential for controlling not only gamma oscillations but also gene expression (Cohen et al., 2016). High frequency firing caused robust Ca^2+^ influx and translocation of Ca^2+^/calmodulin (CaM) to the nucleus, resulting in activity-dependent cAMP response element binding protein (CREB) phosphorylation, synapse-related gene transcription and dendritic branching in a firing frequency- and gamma Ca^2+^/calmodulin-dependent kinase I (CaMKI)- dependent manner. This CREB phosphorylation was tightly related to the effects of HDAC inhibition; HDAC inhibition increases basal transcription machinery and effectively enhances gene transcription levels via the interaction with CREB phosphorylation following neuronal activity (Gräff and Tsai, 2013). One of the epigenetic modification related to HDAC inhibition, NR4a activation was reported to be the synapse-related target of HDAC inhibition and the present study demonstrated that NR4a activation improved deficits in gamma oscillations in PSAPP mice. Thus, fast spiking interneurons play an important role in not only intrinsic excitability- and synapse-related gene expression but also neuronal network activity during cognitive function. More importantly, the novel mechanism of HDAC inhibition for the rescue of activity of fast spiking interneurons in basal and activated state is essential for the improvement of neuronal networks and cognitive improvement.

### Histone Deacetylase Inhibition Acutely Regulated Fast Spiking Neurons Function and Gamma Oscillation

It is assumed that both acute and chronic effects of HDAC inhibition result in the enhancement of synaptic function. Chronic treatment with HDAC inhibitors and the genetic knock down of HDAC improved cognitive function in wild-type and AD model mice via transcriptional mechanisms that led to a long-lasting alteration in gene expression related to synaptic function (Rumbaugh et al., 2015). In contrast, acute treatment with HDAC inhibitors increased LTP in mouse hippocampal slices (Alarcón et al., 2004; Benito et al., 2015) and/or enhanced trafficking of synapse-related proteins onto the cell surface, such as post-synaptic scaffolding proteins and GABA_A_ receptors (Authement et al., 2015). Thus, the underlying mechanisms involved in cognitive function are thought to differ between the acute and chronic effects of HDAC inhibition. Because the present study demonstrated that treatment with HDAC inhibitors for 40 min resulted in an enhancement of fast spiking interneurons and an increase in gamma oscillations, the acute mechanisms might underlie the regulation of the gamma oscillation by HDAC inhibition.

### Gamma Oscillation Was Regulated by Histone Acetylation

Histone acetylation is regulated by the opposing actions of HAT and HDAC (Gräff and Tsai, 2013; Mews et al., 2021). In the present study, focusing on hippocampal gamma oscillations and their important regulator, fast spiking interneurons, we demonstrated a decrease in kainate-induced gamma oscillation in hippocampal slices from PSAPP mice, accompanied with increase of spontaneous activity of fast spiking interneurons in basal state and decrease of kainate-induced activity in activated state. It is the first time that the HDAC inhibitor SAHA was shown to rescue deficits of gamma oscillations, accompanied with improvement of activity of fast spiking interneurons in PSAPP mice in basal and activated state. This effect of SAHA on fast spiking interneurons was different from that of donepezil, which rescued activity of fast spiking interneurons in only basal state. Furthermore, we demonstrated that gamma oscillations are regulated by histone acetylation; enhancement of histone acetylation by HDAC inhibition increased kainate-induced gamma oscillations in both wild-type and PSAPP mice, and conversely a reduction in histone acetylation by HAT inhibition abolished gamma oscillations in wild-type mice. Together with previous studies indicating elevation of HDAC activity in pathological AD (Francis et al., 2009; Ricobaraza et al., 2009; Govindarajan et al., 2011; Gräff and Tsai, 2013; Sen et al., 2015), these data suggest that histone acetylation could be suppressed by elevated HDAC activity, resulting in deficits in gamma oscillations in PSAPP mice. These results indicated a novel mechanism in which HDAC inhibition improved the impairment of gamma oscillations in PSAPP mice by restoring activity of fast spiking interneurons in basal and activated state, potential pathological mechanisms of AD.

### Physiological Implication of Function of Fast Spiking Interneurons During Basal and Activated State for Generation of Gamma Oscillation

In the present study, we found impairment of gamma oscillation in PSAPP mice, accompanied with activity of fast spiking interneurons in basal and activated state. Interestingly, SAHA but not donepezil rescued activity of fast spiking interneurons in activated state in PSAPP mice. Thus, function of fast spiking interneurons regulated by histone acetylation in basal and activated state could be important for regulation of gamma oscillation. The reason why donepezil had no effects on deficit of activity of fast spiking interneurons in activated state but improved deficits of gamma oscillation remains unknown. Based on our result that donepezil alone increased basal gamma oscillation without kainate (Figure 8B), donepezil could influence activity of pyramidal neurons in addition to fast spiking neurons in basal state, accompanied with significant improvement of gamma oscillation via acetylcholine receptors. This increasement of gamma oscillation via acetylcholine is supported by previous studies (Bartos et al., 2007; Spencer et al., 2010). More importantly, it is reported that response of neuronal activity from basal state to activated state could be important for cognitive function (Kaiser and Lutzenberger, 2003; Owen et al., 2013; Espinosa et al., 2019). Together with our result, improvement of gamma oscillation by HDAC inhibition differs from donepezil in terms of state-depend profile. The reversal of gamma oscillation deficits by HDAC inhibition appears to be a potential therapeutic target for treating cognitive impairment in AD patients.

## Data Availability Statement

The original contributions presented in the study are included in the article/Supplementary Material, further inquiries can be directed to the corresponding author/s.

## Ethics Statement

The animal study was reviewed and approved by Animal Care and Use Committee of Shionogi Research Laboratories.

## Author Contributions

KT carried out all electrophysiological studies and wrote the manuscript. KN carried out immunoblotting. MH conceived of the study, participated in its design and helped to prepare the manuscript. KO mainly performed study design, prepared the manuscript and is a corresponding author of this study. All authors read and approved the final manuscript.

## Conflict of Interest

All authors were employed by the company Shionogi & Co., Ltd.

## Publisher’s Note

All claims expressed in this article are solely those of the authors and do not necessarily represent those of their affiliated organizations, or those of the publisher, the editors and the reviewers. Any product that may be evaluated in this article, or claim that may be made by its manufacturer, is not guaranteed or endorsed by the publisher.

## Supplementary Material

Supplementary Figure 1 Kainate-induced activity of fast spiking interneurons was induced by activation of kainate receptors, which was not abolished by block of AMPA and GABA receptors. **(A)** Representative trace indicates that kainate-induced activity of fast spiking interneurons was not abolished by AMPA receptor antagonist GYKI53655 (12.5 μM) and GABA_A_ receptor antagonist bicuculline (10 μM). **(B)** Representative trace indicates that kainate-induced activity of fast spiking interneurons was abolished by kainate/AMPA receptor antagonist CNQX (10 μM).

Supplementary Figure 2 The time course of rescue of kainate-induced gamma oscillation by SAHA was close to that of kainate-induced activity of fast spiking interneurons. **(A)** Representative time course of gamma frequency oscillation (dB) at 20–40 Hz following kainate (200 nM) in slice from PSAPP mice with pretreatment of SAHA (10 μM). **(B)** Representative time course of frequency of action potential in fast spiking interneurons following kainate (200 nM) in slice from PSAPP mice with pretreatment of SAHA (10 μM).

Supplementary Figure 3 SAHA and Donepezil rescued rheobase in fast spiking interneurons of PSAPP mice. **(A)** Rheobase in fast spiking interneurons of PSAPP mice was significantly smaller than that in PS mice (PSAPP mice, *n* = 18 vs PS mice, *n* = 18, *p* < 0.01). This reduction of rheobase in fast spiking interneurons of PSAPP mice was rescued to that in PS mice by SAHA treatment (PSAPP mice treated with SAHA: *n* = 10 vs PS mice: *n* = 18, *p* > 0.05). **(B)** Rheobase in fast spiking interneurons of PSAPP mice was significantly smaller than that in PS mice (PSAPP mice, *n* = 18 vs PS mice, *n* = 18, *p* < 0.01). This reduction of rheobase in fast spiking interneurons of PSAPP mice was rescued to that in PS mice by donepezil treatment (PSAPP mice treated with donepezil: *n* = 10 vs PS mice: *n* = 18, *p* > 0.05).

## References

[B1] Alarcón J. M. Malleret G. Touzani K. Vronskaya S. Ishii S. Kandel E. R. (2004). Chromatin acetylation, memory, and LTP are impaired in CBP+/− mice: a model for the cognitive deficit in Rubinstein-Taybi syndrome and its amelioration. *Neuron* 42 947–959. 10.1016/j.neuron.2004.05.021 15207239

[B2] Andersson R. Johnston A. Fisahn F. (2012). Dopamine D4 receptor activation increases hippocampal gamma oscillations by enhancing synchronization of fast-spiking interneurons. *PLoS One* 7:e40906. 10.1371/journal.pone.0040906 22815864 PMC3398948

[B3] Authement M. E. Kodangattil J. N. Gouty S. Rusnak M. Symes A. J. Cox B. M. (2015). Histone deacetylase inhibition rescues maternal deprivation-induced GABAergic metaplasticity through restoration of AKAP Signaling. *Neuron* 86 1240–1252. 10.1016/j.neuron.2015.05.024 26050042

[B4] Bartos M. Vida I. Jonas P. (2007). Synaptic mechanisms of synchronized gamma oscillations in inhibitory interneuron networks. *Nat. Rev. Neurosci.* 8 45–56. 17180162 10.1038/nrn2044

[B5] Benito E. Urbanke H. Ramachandran B. Barth J. Halder R. Awasthi A. (2015). HDAC inhibitor-dependent transcriptome and memory reinstatement in cognitive decline models. *J. Clin. Invest.* 125 3572–3584. 10.1172/JCI79942 26280576 PMC4588238

[B6] Birks J. S. Harvey R. (2018). Donepezil for dementia due to Alzheimer’s disease. *Cochrane Database Syst. Rev.* 6:CD001190. 10.1002/14651858.CD001190 12917900

[B7] Born H. A. Kim J. Y. Savjani R. R. Das P. Dabaghian Y. A. Guo Q. (2014). Genetic suppression of transgenic APP rescues Hypersynchronous network activity in a mouse model of Alzeimer’s disease. *J. Neurosci.* 34 3826–3840. 10.1523/JNEUROSCI.5171-13.2014 24623762 PMC3951689

[B8] Buzsaki G. Wang X. J. (2012). Mechanisms of gamma oscillations. *Annu. Rev. Neurosci.* 35 203–225. 22443509 10.1146/annurev-neuro-062111-150444 PMC4049541

[B9] Cardin J. A. Carlén M. Meletis K. Knoblich U. Zhang F. Deisseroth K. (2009). Driving fast-spiking cells induces gamma rhythm and controls sensory responses. *Nature* 459 663–667. 10.1038/nature08002 19396156 PMC3655711

[B10] Chung H. Park K. Jang H. J. Kohl M. M. Kwag J. (2020). Dissociation of somatostatin and parvalbumin interneurons circuit dysfunctions underlying hippocampal theta and gamma oscillations impaired by amyloid b oligomers in vivo. *Brain Struct. Funct.* 225 935–954. 10.1007/s00429-020-02044-3 32107637 PMC7166204

[B11] Cohen S. M. Ma H. Kuchibhotla K. V. Watson B. O. Buzsáki G. Froemke R. C. (2016). Excitation-transcription coupling in parvalbumin-positive interneurons employs a novel CaM kinase-dependent pathway distinct from excitatory neurons. *Neuron* 90 292–307. 10.1016/j.neuron.2016.03.001 27041500 PMC4866871

[B12] Espinosa N. Alonso A. Lara-Vasquez A. Fuentealba P. (2019). Basal forebrain somatostatin cells differentially regulate local gamma oscillations and functionally segregate motor and cognitive circuits. *Sci. Rep.* 9:2570. 10.1038/s41598-019-39203-4 30796293 PMC6384953

[B13] Etter G. Veldt S. Manseau F. Zarrinkoub I. Doppia E. T. Williams S. (2019). Optogenetic gamma stimulation rescues memory impairments in an Alzheimer’s disease mouse model. *Nat. Commun.* 10:5322. 10.1038/s41467-019-13260-9 31757962 PMC6876640

[B14] Fell J. Klaver P. Lehnertz K. Grunwald T. Schaller C. Elger C. E. (2001). Human memory formation is accompanied by rhinal-hippocampal coupling and decoupling. *Nat. Neurosci.* 4 1259–1264. 11694886 10.1038/nn759

[B15] Fisahn A. Contractor A. Traub R. D. Buhl E. H. Heinemann S. F. McBain C. J. (2004). Distinct roles for the kainate receptor subunits GluR5 and GluR6 in kainate-induced hippocampal gamma oscillations. *J. Neurosci.* 24 9658–9668. 10.1523/JNEUROSCI.2973-04.2004 15509753 PMC6730151

[B16] Francis Y. I. Fà M. Ashraf H. Zhang H. Staniszewski A. Latchman D. S. (2009). Dysregulation of histone acetylation in the APP/PS1 mouse model of Alzheimer’s disease. *J. Alzheimers Dis.* 18 131–139. 19625751 10.3233/JAD-2009-1134 PMC8962655

[B17] Govindarajan N. Agis-Balboa R. C. Walter J. Sananbenesi F. Fischer A. (2011). Sodium butyrate improves memory function in an Alzheimer’s disease mouse model when administered at an advanced stage of disease progression. *J. Alzheimers Dis.* 26 187–197. 10.3233/JAD-2011-110080 21593570

[B18] Gräff J. Tsai L. H. (2013). Histone acetylation: molecular mnemonics on the chromatin. *Nat. Rev. Neurosci.* 14 97–111. 23324667 10.1038/nrn3427

[B19] Guan J. S. Haggarty S. J. Giacometti E. Dannenberg J. H. Joseph N. Gao J. (2009). HDAC2 negatively regulates memory formation and synaptic plasticity. *Nature* 459 55–60. 19424149 10.1038/nature07925 PMC3498958

[B20] Guan Z. Giustetto M. Lomvardas S. Kim J. H. Miniaci M. C. Schwartz J. H. (2002). Integration of long-term-memory-related synaptic plasticity involves bidirectional regulation of gene expression and chromatin structure. *Cell* 111 483–493. 10.1016/s0092-8674(02)01074-7 12437922

[B21] Hartley D. M. Walsh D. M. Ye C. P. Diehl T. Vasquez S. Vassilev P. M. (1999). Protofibrillar intermediates of amyloid β-protein induce acute electrophysiological changes and progressive neurotoxicity in cortical neurons. *J. Neurosci.* 19 8876–8884. 10.1523/JNEUROSCI.19-20-08876.1999 10516307 PMC6782787

[B22] Hawk J. D. Bookout A. L. Poplawski S. G. Bridi M. Rao A. J. Sulewski M. E. (2012). NR4A nuclear receptors support memory enhancement by histone deacetylase inhibitors. *J. Clin. Invest.* 122 3593–3602. 22996661 10.1172/JCI64145 PMC3461922

[B23] Hazra A. Gu F. Aulakh A. Berridge C. Eriksen J. L. Ziburkus J. (2013). Inhibitory neuron and hippocampal circuit dysfunction in an aged mouse model of Alzheimer’s Disease. *PLoS One* 8:e64318. 10.1371/journal.pone.0064318 23691195 PMC3656838

[B24] Herrmann C. S. Demiralp T. (2005). Human EEG gamma oscillations in neuropsychiatric disorders. *Clin. Neurophysiol.* 116 2719–2733. 16253555 10.1016/j.clinph.2005.07.007

[B25] Herrmann C. S. Fründ I. Lenz D. (2010). Human gamma-band activity: a review on cognitive and behavioral correlates and network models. *Neurosci. Biobehav. Rev.* 34 981–992. 10.1016/j.neubiorev.2009.09.001 19744515

[B26] Hsiao K. Chapman P. Nilsen S. Eckman C. Harigaya Y. Younkin S. (1996). Correlative memory deficits, Aβ elevation, and amyloid plaques in transgenic mice. *Science* 274 99–102. 10.1126/science.274.5284.99 8810256

[B27] Hu H. Gan J. Jonas P. (2014). Fast-spiking, parvalbumin^+^ GABAergic interneurons: from cellular design to microcircuit function. *Science* 345:1255263. 10.1126/science.1255263 25082707

[B28] Igarashi K. M. Lu L. Colgin L. L. Moser M. B. Moser E. I. (2014). Coordination of entorhinal-hippocampal ensemble activity during associative learning. *Nature* 510 143–147. 10.1038/nature13162 24739966

[B29] Iwasaki S. Sasaki T. Ikegaya Y. (2021). Hippocampal beta oscillations predict mouse object-location associative memory performance. *Hippocampus* 31 503–511. 10.1002/hipo.23311 33556218

[B30] Joosen M. J. van Helden H. P. (2007). Correlations between acetylcholinesterase inhibition, acetylcholine levels and EEG changes during perfusion with neostigmine and N6-cyclopentyladenosine in rat brain. *Eur. J. Pharmacol.* 555 122–128. 10.1016/j.ejphar.2006.10.006 17113068

[B31] Kaiser J. Lutzenberger W. (2003). Induced gamma-band activity and human brain function. *Neuroscientist* 9 475–484. 14678580 10.1177/1073858403259137

[B32] Kamenetz F. Tomita T. Hsieh H. Seabrook G. Borchelt D. Iwatsubo T. (2003). APP processing and synaptic function. *Neuron* 37 925–937. 12670422 10.1016/s0896-6273(03)00124-7

[B33] Kilkenny C. Browne W. Cuthill I. C. Emerson M. Altman D. G. (2010). Animal research: reporting in vivo experiments: the ARRIVE guidelines. *Br. J. Pharmacol.* 160 1577–1579. 20649561 10.1111/j.1476-5381.2010.00872.x PMC2936830

[B34] Kispersky T. J. Caplan J. S. Marder E. (2012). Increase in sodium conductance decreases firing rate and gain in model neurons. *J. Neurosci.* 32 10995–11004. 22875933 10.1523/JNEUROSCI.2045-12.2012 PMC3427781

[B35] Kodama D. Ono H. Tanabe M. (2011). Increased hippocampal glycine uptake and cognitive dysfunction after peripheral nerve injury. *Pain* 152 809–817. 10.1016/j.pain.2010.12.029 21295405

[B36] Lewis D. A. Curley A. A. Glausier J. R. Volk D. W. (2012). Cortical parvalbumin interneurons and cognitive dysfunction in schizophrenia. *Trends Neurosci.* 35 57–67. 22154068 10.1016/j.tins.2011.10.004 PMC3253230

[B37] Marin O. (2012). Interneuron dysfunction in psychiatric disorders. *Nat. Rev. Neurosci.* 13 107–120. 22251963 10.1038/nrn3155

[B38] Matsushita Y. Araki K. Omotuyi O. Mukae T. Ueda H. (2013). HDAC inhibitors restore C-fibre sensitivity in experimental neuropathic pain model. *Br. J. Pharmacol.* 170 991–998. 10.1111/bph.12366 24032674 PMC3949648

[B39] McGrath J. Drummond G. McLachlan E. Kilkenny C. Wainwright C. (2010). Guidelines for reporting experiments involving animals: the ARRIVE guidelines. *Br. J. Pharmacol.* 160 1573–1576. 20649560 10.1111/j.1476-5381.2010.00873.x PMC2936829

[B40] Mews P. Calipari E. S. Day J. Lobo M. K. Bredy T. Abel T. (2021). From circuits to chromatin: the emerging role of epigenetics in mental health. *J. Neurosci.* 41 873–882. 10.1523/JNEUROSCI.1649-20.2020 33446519 PMC7880276

[B41] Mitchell S. J. Silver R. A. (2003). Shunting inhibition modulates neuronal gain during synaptic excitation. *Neuron* 38 433–445. 12741990 10.1016/s0896-6273(03)00200-9

[B42] Nakano Y. Kondoh G. Kudo T. Imaizumi K. Kato M. Miyazaki J. (1999). Accumulation of murine amyloidβ42 in a gene-dosage-dependent manner in PS1 ‘knock-in’ mice. *Eur. J. Neurosci.* 11 2577–2581. 10383647 10.1046/j.1460-9568.1999.00698.x

[B43] Ohnishi T. Yanazawa M. Sasahara T. Kitamura Y. Hiroaki H. Fukazawa Y. (2015). Na, K-ATPase α3 is a death target of Alzheimer patient amyloid-β assembly. *Proc. Natl. Acad. Sci. U. S. A.* 112 E4465–E4474. 26224839 10.1073/pnas.1421182112 PMC4538662

[B44] Owen S. Tuncdemir S. N. Bader P. L. Tirko N. N. Fishell G. Tsien R. W. (2013). Oxytocin enhances hippocampal spike transmission by modulating fast-spiking interneurons. *Nature* 500 458–462. 10.1038/nature12330 23913275 PMC5283693

[B45] Palop J. J. Mucke L. (2010). Amyloid-β-induced neuronal dysfunction in Alzheimer’s disease: from synapses toward neural networks. *Nat. Neurosci.* 13 812–818. 10.1038/nn.2583 20581818 PMC3072750

[B46] Petrushanko I. Y. Mitkevich V. A. Anashkina A. A. Adzhubei A. A. Burnysheva K. M. Lakunina V. A. (2016). Direct interaction of beta-amyloid with Na,K-ATPase as a putative regulator of the enzyme function. *Sci. Rep.* 6:27738. 10.1038/srep27738 27296892 PMC4906314

[B47] Phillips J. M. Ehrlichman R. S. Siegel S. J. (2007). Mecamylamine blocks nicotine induced enhancement of the P20 auditory event-related potential and evoked gamma. *Neuroscience* 144 1314–1323. 10.1016/j.neuroscience.2006.11.003 17184927 PMC1868669

[B48] Ricobaraza A. Cuadrado-Tejedor M. Pérez-Mediavilla A. Frechilla D. Del Río J. García-Osta A. (2009). Phenylbutyrate ameliorates cognitive deficit and reduces tau pathology in an Alzheimer’s disease mouse model. *Neuropsychopharmacology* 34 1721–1732. 10.1038/npp.2008.229 19145227

[B49] Rumbaugh G. Sillivan S. E. Ozkan E. D. Rojas C. S. Hubbs C. R. Aceti M. (2015). Pharmacological Selectivity Within Class I Histone Deacetylases Predicts Effects on Synaptic Function and Memory Rescue. *Neuropsychopharmacology* 40 2307–2316. 25837283 10.1038/npp.2015.93 PMC4538358

[B50] Salkoff D. B. Zagha E. Yüzgeç Ö McCormick D. A. (2015). Synaptic Mechanisms of Tight Spike Synchrony at Gamma Frequency in Cerebral Cortex. *J. Neurosci.* 35 10236–10251. 26180200 10.1523/JNEUROSCI.0828-15.2015 PMC4502264

[B51] Sederberg P. B. Schulze-Bonhage A. Madsen J. R. Bromfield E. B. McCarthy D. C. Brandt A. (2007). Hippocampal and neocortical gamma oscillations predict memory formation in humans. *Cereb. Cortex* 17 1190–1196. 16831858 10.1093/cercor/bhl030

[B52] Selkoe D. J. (2002). Alzheimer’s disease is a synaptic failure. *Science* 298 789–791. 12399581 10.1126/science.1074069

[B53] Sen A. Nelson T. J. Alkon D. L. (2015). ApoE4 and Aβ oligomers reduce BDNF expression via HDAC nuclear translocation. *J. Neurosci.* 35 7538–7551. 10.1523/JNEUROSCI.0260-15.2015 25972179 PMC6705431

[B54] Spencer J. P. Middleton L. J. Davies C. H. (2010). Investigation into the efficacy of the acetylcholinesterase inhibitor, donepezil, and novel procognitive agents to induce gamma oscillations in rat hippocampal slices. *Neuropharmacology* 59 437–443. 10.1016/j.neuropharm.2010.06.005 20600173

[B55] Tao W. Chen Q. Wang L. Zhou W. Wang Y. Zhang Z. (2015). Brainstem brain-derived neurotrophic factor signaling is required for histone deacetylase inhibitor-induced pain relief. *Mol. Pharmacol.* 87 1035–1041. 10.1124/mol.115.098186 25852071

[B56] Tombaugh G. C. Rowe W. B. Rose G. M. (2005). The slow afterhyperpolarization in hippocampal CA1 neurons covaries with spatial learning ability in aged Fisher 344 rats. *J. Neurosci.* 25 2609–2616. 10.1523/JNEUROSCI.5023-04.2005 15758171 PMC6725166

[B57] Uchida H. Ma L. Ueda H. (2010). Epigenetic gene silencing underlies C-fiber dysfunctions in neuropathic pain. *J. Neurosci.* 30 4806–4814. 10.1523/JNEUROSCI.5541-09.2010 20357131 PMC6632306

[B58] Vecsey C. G. Hawk J. D. Lattal K. M. Stein J. M. Fabian S. A. Attner M. A. (2007). Histone deacetylase inhibitors enhance memory and synaptic plasticity via CREB: CBP-dependent transcriptional activation. *J. Neurosci.* 27 6128–6140. 10.1523/JNEUROSCI.0296-07.2007 17553985 PMC2925045

[B59] Verheugen J. H. Fricker D. Miles R. (1999). Noninvasive measurements of the membrane potential and GABAergic action in hippocampal interneurons. *J. Neurosci.* 19 2546–2555. 10.1523/JNEUROSCI.19-07-02546.1999 10087068 PMC6786065

[B60] Verret L. Mann E. O. Hang G. B. Barth A. M. I. Cobos I. Ho K. (2012). Inhibitory interneuron deficit links altered network activity and cognitive dysfunction in Alzheimer model. *Cell* 149 708–721. 22541439 10.1016/j.cell.2012.02.046 PMC3375906

[B61] Walls A. B. Eyjolfsson E. M. Smeland O. B. Nilsen L. H. Schousboe I. Schousboe A. (2011). Knockout of GAD65 has major impact on synaptic GABA synthesized from astrocyte-derived glutamine. *J. Cereb. Blood Flow Metab.* 31 494–503. 10.1038/jcbfm.2010.115 20664610 PMC3049505

[B62] Walsh D. M. Selkoe D. J. (2007). Aβ oligomers - a decade of discovery. *J. Neurochem.* 101 1172–1184. 17286590 10.1111/j.1471-4159.2006.04426.x

[B63] Whitehead A. Perdomo C. Pratt R. D. Birks J. Wilcock G. K. Evans J. G. (2004). Donepezil for the symptomatic treatment of patients with mild to moderate Alzheimer’s disease: a meta-analysis of individual patient data from randomised controlled trials. *Int. J. Geriatr. Psychiatry* 19 624–633. 10.1002/gps.1133 15254918

[B64] Yamamoto J. Suh J. Takeuchi D. Tonegawa S. (2014). Successful execution of working memory linked to synchronized high-frequency gamma oscillations. *Cell* 157 845–857. 10.1016/j.cell.2014.04.009 24768692

[B65] Ye C. P. Selkoe D. J. Hartley D. M. (2003). Protofibrils of amyloid β-protein inhibit specific K^+^ currents in neocortical cultures. *Neurobiol. Dis.* 13 177–190. 10.1016/s0969-9961(03)00068-8 12901832

[B66] Zhang Z. Cai Y. Q. Zou F. Bie B. Pan Z. Z. (2011). Epigenetic suppression of GAD65 expression mediates persistent pain. *Nat. Med.* 17 1448–1455. 10.1038/nm.2442 21983856 PMC3210928

