# Supplementary figures and images for: Histone Deacetylase Inhibitor Improves the Dysfunction of Hippocampal Gamma Oscillations and Fast Spiking Interneurons in Alzheimer’s Disease Model Mice
Source: Front Mol Neurosci. 2021 Dec 23;14:782206. doi: 10.3389/fnmol.2021.782206 (PMC8751405; doi:10.3389/fnmol.2021.782206)

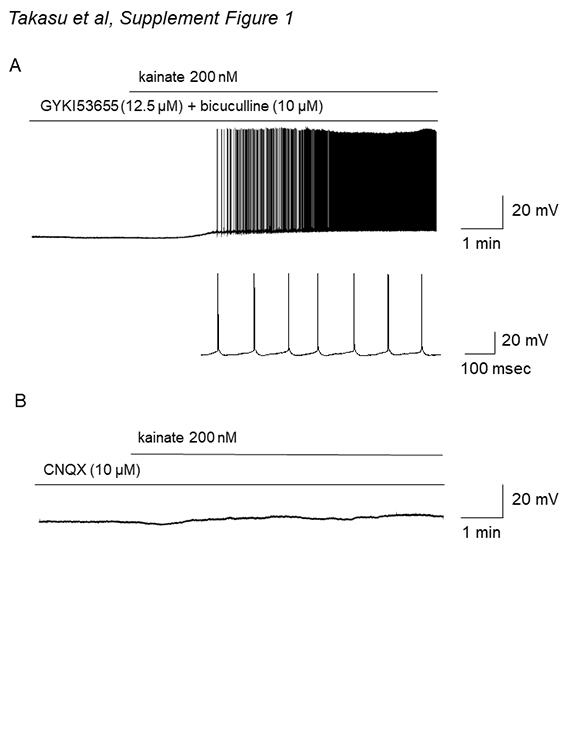

Supplement: Supplementary Figure 1 — Kainate-induced activity of fast spiking interneurons was induced by activation of kainate receptors, which was not abolished by block of AMPA and GABA receptors. (A) Representative trace indicates that kainate-induced activity of fast spiking interneurons was not abolished by AMPA receptor antagonist GYKI53655 (12.5 μM) and GABAA receptor antagonist bicuculline (10 μM). (B) Representative trace indicates that kainate-induced activity of fast spiking interneurons was abolished by kainate/AMPA receptor antagonist CNQX (10 μM). [file Image_1.tif]

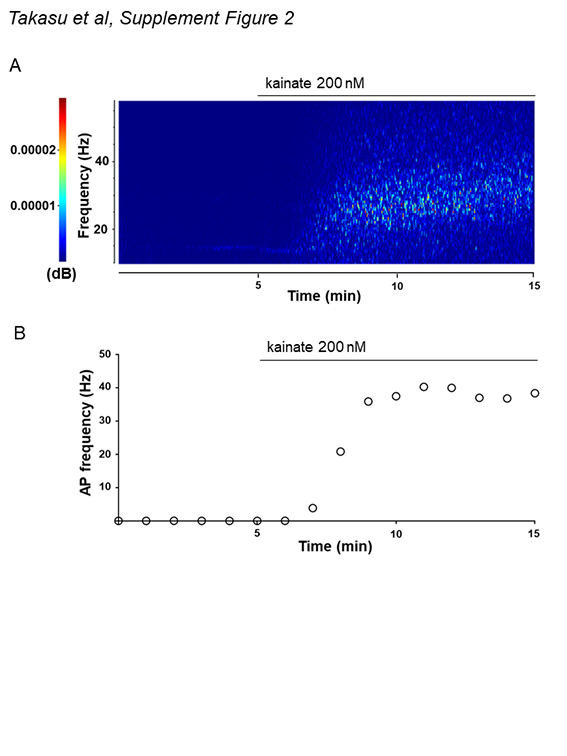

Supplement: Supplementary Figure 2 — The time course of rescue of kainate-induced gamma oscillation by SAHA was close to that of kainate-induced activity of fast spiking interneurons. (A) Representative time course of gamma frequency oscillation (dB) at 20–40 Hz following kainate (200 nM) in slice from PSAPP mice with pretreatment of SAHA (10 μM). (B) Representative time course of frequency of action potential in fast spiking interneurons following kainate (200 nM) in slice from PSAPP mice with pretreatment of SAHA (10 μM). [file Image_2.tif]

Takasu et al, Supplement Figure 3

A

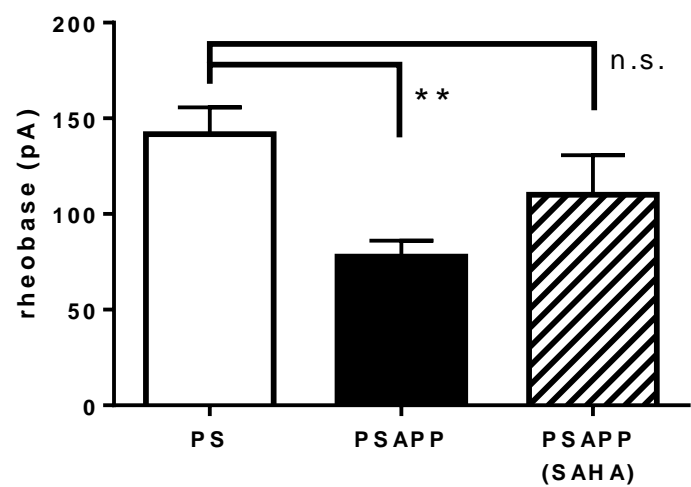

B

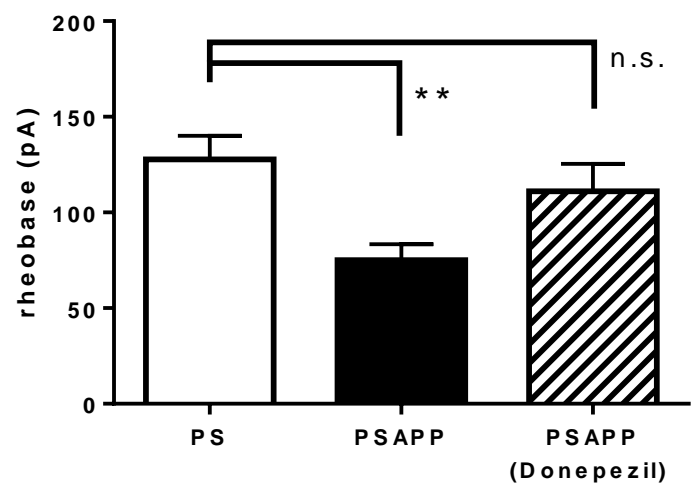

Supplement: Supplementary Figure 3 — SAHA and Donepezil rescued rheobase in fast spiking interneurons of PSAPP mice. (A) Rheobase in fast spiking interneurons of PSAPP mice was significantly smaller than that in PS mice (PSAPP mice, n = 18 vs PS mice, n = 18, p < 0.01). This reduction of rheobase in fast spiking interneurons of PSAPP mice was rescued to that in PS mice by SAHA treatment (PSAPP mice treated with SAHA: n = 10 vs PS mice: n = 18, p > 0.05). (B) Rheobase in fast spiking interneurons of PSAPP mice was significantly smaller than that in PS mice (PSAPP mice, n = 18 vs PS mice, n = 18, p < 0.01). This reduction of rheobase in fast spiking interneurons of PSAPP mice was rescued to that in PS mice by donepezil treatment (PSAPP mice treated with donepezil: n = 10 vs PS mice: n = 18, p > 0.05). [file Image_3.pdf]
